# Supplementary material for: Comparative efficacy and complication rates after local treatment for cervical intraepithelial neoplasia and stage 1a1 cervical cancer: protocol for a systematic review and network meta-analysis from the CIRCLE Group
Source: BMJ Open. 2019 Aug 2;9(8):e028008. doi: 10.1136/bmjopen-2018-028008 (PMC6687014; doi:10.1136/bmjopen-2018-028008)
Supplement: Supplementary data [file bmjopen-2018-028008supp002.pdf]

Supplementary File 2: Search algorithms

Medline Ovid RCT only

1 exp Cervical Intraepithelial Neoplasia/
2 CIN.mp.
3 (cervi* and (intraepithel* or epithel*)).mp.
4 (cervi* and dysplasia).mp.
5 (cervi* and carcinoma in situ).mp.
6 (cervi* and cancer in situ).mp.
7 (cervi* and (precancer* or pre-cancer*)).mp.
8 1 or 2 or 3 or 4 or 5 or 6 or 7
9 surgery.fs.
10 exp Gynecologic Surgical Procedures/
11 (surg* or ablat* or excis* or cryotherapy or laser* or cone or conisation or biopsy or transformation zone or LLETZ or LEEP).mp.
12 9 or 10 or 11
13 8 and 12
14 randomized controlled trial.pt.
15 controlled clinical trial.pt.
16 randomized.ab.
17 placebo.ab.
18 clinical trials as topic.sh.
19 randomly.ab.
20 trial.ti.
21 14 or 15 or 16 or 17 or 18 or 19 or 20
22 13 and 21

Medline Ovid NON RCT only

1. exp Cervical Intraepithelial Neoplasia/
2. CIN.mp. [mp=title, abstract, original title, name of substance word, subject heading word, keyword heading word, protocol supplementary concept word, rare disease supplementary concept word, unique identifier, synonyms]
3. (cervi* and (intraepithel* or epithel*)).mp. [mp=title, abstract, original title, name of substance word, subject heading word, keyword heading word, protocol supplementary concept word, rare disease supplementary concept word, unique identifier, synonyms]
4. (cervi* and dysplasia).mp. [mp=title, abstract, original title, name of substance word, subject heading word, keyword heading word, protocol supplementary concept word, rare disease supplementary concept word, unique identifier, synonyms]

5. (cervi* and carcinoma in situ).mp. [mp=title, abstract, original title, name of substance word, subject heading word, keyword heading word, protocol supplementary concept word, rare disease supplementary concept word, unique identifier, synonyms]
6. (cervi* and cancer in situ).mp. [mp=title, abstract, original title, name of substance word, subject heading word, keyword heading word, protocol supplementary concept word, rare disease supplementary concept word, unique identifier, synonyms]
7. (cervi* and (precancer* or pre-cancer*)).mp. [mp=title, abstract, original title, name of substance word, subject heading word, keyword heading word, protocol supplementary concept word, rare disease supplementary concept word, unique identifier, synonyms]
8. 1 or 2 or 3 or 4 or 5 or 6 or 7
9. surgery.fs.
10. exp Gynecologic Surgical Procedures/
11. (surg* or ablat* or excis* or cryotherapy or laser* or cone or conisation or biopsy or transformation zone or LLETZ or LEEP).mp. [mp=title, abstract, original title, name of substance word, subject heading word, keyword heading word, protocol supplementary concept word, rare disease supplementary concept word, unique identifier, synonyms]
12. 9 or 10 or 11
13. 8 and 12
14. randomized controlled trial.pt.
15. controlled clinical trial.pt.
16. randomized.ab.
17. placebo.ab.
18. clinical trials as topic.sh.
19. randomly.ab.
20. trial.ti.
21. groups.ab.
22. exp cohort studies/
23. exp case-control studies/
24. (cohort* or prospective* or retrospective* or (case* and (control* or series))).mp.
25. 14 or 15 or 16 or 17 or 18 or 19 or 20 or 21 or 22 or 23 or 24
26. (animals not (humans and animals)).sh.
27. 25 not 26
28. 13 and 27

Embase Ovid RCT only

1 exp Uterine Cervix Carcinoma in Situ/

2 CIN.mp.
3 (cervi* and (intraepithel* or epithel*)).mp.
4 (cervi* and dysplasia).mp.
5 (cervi* and carcinoma in situ).mp.
6 (cervi* and cancer in situ).mp.
7 (cervi* and (precancer* or pre-cancer*)).mp.
8 1 or 2 or 3 or 4 or 5 or 6 or 7
9 su.fs.
10 exp gynecologic surgery/
11 (surg* or ablat* or excis* or cryotherapy or laser* or cone or conisation or biopsy or transformation zone or LLETZ or LEEP).mp.
12 9 or 10 or 11
13 8 and 12
14 crossover procedure/
15 double-blind procedure/
16 randomized controlled trial/
17 single-blind procedure/
18 random*.mp.
19 factorial*.mp.
20 (crossover* or cross over* or cross-over*).mp.
21 placebo*.mp.
22 (double* adj blind*).mp.
23 (singl* adj blind*).mp.
24 assign*.mp.
25 allocat*.mp.
26 volunteer*.mp.
27 14 or 15 or 16 or 17 or 18 or 19 or 20 or 21 or 22 or 23 or 24 or 25 or 26
28 13 and 27

Embase Ovid All Studies

1. exp Uterine Cervix Carcinoma in Situ/
2. CIN.mp. [mp=title, abstract, heading word, drug trade name, original title, device manufacturer, drug manufacturer, device trade name, keyword, floating subheading word]
3. (cervi* and (intraepithel* or epithel*)).mp. [mp=title, abstract, heading word, drug trade name, original title, device manufacturer, drug manufacturer, device trade name, keyword, floating subheading word]

4. (cervi* and dysplasia).mp. [mp=title, abstract, heading word, drug trade name, original title, device manufacturer, drug manufacturer, device trade name, keyword, floating subheading word]
5. (cervi* and carcinoma in situ).mp. [mp=title, abstract, heading word, drug trade name, original title, device manufacturer, drug manufacturer, device trade name, keyword, floating subheading word]
6. (cervi* and cancer in situ).mp. [mp=title, abstract, heading word, drug trade name, original title, device manufacturer, drug manufacturer, device trade name, keyword, floating subheading word]
7. (cervi* and (precancer* or pre-cancer*)).mp. [mp=title, abstract, heading word, drug trade name, original title, device manufacturer, drug manufacturer, device trade name, keyword, floating subheading word]
8. 1 or 2 or 3 or 4 or 5 or 6 or 7
9. su.fs.
10. exp gynecologic surgery/
11. (surg* or ablat* or excis* or cryotherapy or laser* or cone or conisation or biopsy or transformation zone or LLETZ or LEEP).mp.
12. 9 or 10 or 11
13. 8 and 12
14. exp controlled clinical trial/
15. randomized.ab.
16. randomly.ab.
17. trial.ab.
18. groups.ab.
19. exp cohort analysis/
20. cohort*.mp.
21. exp retrospective study/
22. exp prospective study/
23. (case* and series).mp.
24. 14 or 15 or 16 or 17 or 18 or 19 or 20 or 21 or 22 or 23
25. 13 and 24

CENTRAL

#1 MeSH descriptor Cervical Intraepithelial Neoplasia explode all trees
#2 CIN
#3 cervi* and (intraepithel* or epithel*)
#4 cervi* and dysplasia
#5 cervi* and carcinoma in situ
#6 cervi* and cancer in situ
#7 cervi* and (precancer* or pre-cancer*)
#8 (#1 OR #2 OR #3 OR #4 OR #5 OR #6 OR #7)

#9 Any MeSH descriptor with qualifier(s): [Surgery - SU]
#10 MeSH descriptor: [Gynecologic Surgical Procedures] explode all trees
#11 surg* or ablat* or excis* or cryotherapy or laser* or cone or conisation or biopsy or transformation zone or LLETZ or LEEP
#12 #9 or #10 or #11
#13 #8 and #12
