# Supplementary material for: Comparative efficacy and complication rates after local treatment for cervical intraepithelial neoplasia and stage 1a1 cervical cancer: protocol for a systematic review and network meta-analysis from the CIRCLE Group
Source: BMJ Open. 2019 Aug 2;9(8):e028008. doi: 10.1136/bmjopen-2018-028008 (PMC6687014; doi:10.1136/bmjopen-2018-028008)
Supplement: Author's manuscript [file bmjopen-2018-028008.draft_revisions.pdf]

BMJ Open is committed to open peer review. As part of this commitment we make the peer review history of every article we publish publicly available.

When an article is published we post the peer reviewers' comments and the authors' responses online. We also post the versions of the paper that were used during peer review. These are the versions that the peer review comments apply to.

The versions of the paper that follow are the versions that were submitted during the peer review process. They are not the versions of record or the final published versions. They should not be cited or distributed as the published version of this manuscript.

BMJ Open is an open access journal and the full, final, typeset and author-corrected version of record of the manuscript is available on our site with no access controls, subscription charges or pay-per-view fees (http://bmjopen.bmj.com).

If you have any questions on BMJ Open's open peer review process please email info.bmjopen@bmj.com

BMJ Open

Comparative efficacy and complication rates after local treatment for cervical intra-epithelial neoplasia and stage 1a1 cervical cancer: protocol for a systematic review and network meta-analysis from the CIRCLE Group

Journal:	*BMJ Open*
Manuscript ID	bmjopen-2018-028008
Article Type:	Protocol
Date Submitted by the Author:	18-Nov-2018
Complete List of Authors:	Athanasiou, Antonios; Institute of Reproductive and Developmental Biology, Department of Surgery & Cancer, Faculty of Medicine, Imperial College London; Imperial College Healthcare NHS Trust<br>Veroniki, Areti Angeliki; Institute of Reproductive and Developmental Biology, Department of Surgery & Cancer, Faculty of Medicine, Imperial College London; Panepistimio Ioanninon, School of Education, Department of Primary Education<br>Eftimiou, Orestis; University of Bern, Institute of Social and Preventive Medicine (ISPM)<br>Kalliala, Ilkka; Institute of Reproductive and Developmental Biology, Department of Surgery & Cancer, Faculty of Medicine, Imperial College London; University of Helsinki and Helsinki University Hospital, Department of Obstetrics and Gynaecology<br>Naci, Huseyin; London School of Economics, Department of Health Policy<br>Lever, Sarah; Institute of Reproductive and Developmental Biology, Department of Surgery & Cancer, Faculty of Medicine, Imperial College London; Imperial College Healthcare NHS Trust<br>Paraskevaidi, Maria; Institute of Reproductive and Developmental Biology, Department of Surgery & Cancer, Faculty of Medicine, Imperial College London<br>Martin-Hirsch, Pierre; Lancashire Teaching Hospitals NHS Foundation Trust, Department of Gynaecologic Oncology<br>Bennett, Philip; Institute of Reproductive and Developmental Biology, Department of Surgery & Cancer, Faculty of Medicine, Imperial College London; Imperial College Healthcare NHS Trust<br>Paraskevaidis, Evangelos; University of Ioannina and University Hospital of Ioannina, Department of Obstetrics & Gynaecology; Imperial College Healthcare NHS Trust<br>Salanti, Georgia; University of Bern, Institute of Social and Preventive Medicine (ISPM)<br>Kyrgiou, Maria; Institute of Reproductive and Developmental Biology, Department of Surgery & Cancer, Faculty of Medicine, Imperial College London; Imperial College Healthcare NHS Trust
Keywords:	"Cervical Intraepithelial Neoplasia/surgery"[Mesh], "Cervical Intraepithelial Neoplasia/therapy"[Mesh], "Treatment Outcome"[Mesh], "Recurrence"[Mesh], "Conization/adverse effects"[Mesh], "Ablation Techniques/adverse effects"[Mesh]

Comparative efficacy and complication rates after local treatment for cervical intra-epithelial neoplasia and stage 1a1 cervical cancer: protocol for a systematic review and network meta-analysis from the CIRCLE Group

Antonios Athanasiou[1,2], Areti Angeliki Veroniki[1,3], Orestis Efthimiou[4], Ilkka Kalliala[1,5], Huseyin Naci[6], Sarah Lever[1,2], Maria Paraskevaidi[1], Pierre Martin-Hirsch[7], Phillip Bennett[1,2], Evangelos Paraskevaidis[2,8], Georgia Salanti[4], Maria Kyrgiou[1,2]

[1]Institute of Reproductive and Developmental Biology, Department of Surgery & Cancer, Faculty of Medicine, Imperial College London, UK
[2]Imperial Healthcare NHS Trust, London, UK
[3]Department of Primary Education, School of Education, University of Ioannina, Greece
[4]Institute of Social and Preventive Medicine (ISPM), University of Bern, Finkenhubelweg 11, 3012 Bern, Switzerland
[5]Department of Obstetrics and Gynaecology, University of Helsinki and Helsinki University Hospital, Finland
[6]Department of Health Policy, London School of Economics & Political Science, UK
[7]Department of Gynaecologic Oncology, Lancashire Teaching Hospitals, Preston, UK
[8]Department of Obstetrics & Gynaecology, University of Ioannina and University Hospital of Ioannina, Greece

AA: a.athanasiou@imperial.ac.uk; AAV: a.veroniki@imperial.ac.uk; OE: orestis.efthimiou@ispm.unibe.ch; IK: i.kalliala@imperial.ac.uk; HN: h.naci@lse.ac.uk; SL: s.lever@imperial.ac.uk; MP: m.paraskevaidi@imperial.ac.uk; PMH: pierre.martin-hirsch@lthtr.nhs.uk; PB: p.bennett@imperial.ac.uk; EP: eparaske@cc.uoi.gr; GS: georgia.salanti@ispm.unibe.ch; MK: m.kyrgiou@imperial.ac.uk

Corresponding author:
Maria Kyrgiou, MSc, PhD, MRCOG
3rd Floor, IRDB, Department of Surgery & Cancer
Imperial College London, Hammersmith Campus
Du Cane Road, W12 0NN, London
Email: m.kyrgiou@imperial.ac.uk - Tel: +44 2075942177

Word Count: 3010

For peer review only

ABSTRACT

Introduction: Local treatments for cervical intra-epithelial neoplasia (CIN) and micro-invasive disease remove or ablate a cone-shaped part of the uterine cervix containing the abnormal cells. A trend towards less radical techniques has raised concerns that this may adversely impact the rates of precancerous and cancerous recurrence. However, there has been no strong evidence to support such claims. We hereby describe a protocol of a systematic review and network meta-analysis that will update the evidence and compare all relevant treatments in terms of efficacy and complications.

Methods and Analysis: Literature searches in electronic databases or trial registries will identify published and unpublished randomised controlled trials (RCTs) and cohort studies comparing the efficacy and complications amongst different excisional and ablative techniques. The excisional techniques include cold knife, laser or fischer cone, large loop or needle excision of the transformation zone and the ablative radical point diathermy, cryotherapy, cold coagulation or laser ablation. The primary outcome will be high-grade treatment failure rates, defined as recurrent/residual histologically-proven CIN2 or worse, while secondary outcomes will include treatment failure rates at CIN1+, HPV positivity rates, abnormal cytology and/or histology rates, involved margins rates, and rates of bleeding, inadequate colposcopy and cervical stenosis. We will assess the risk of bias in RCTs and observational studies using tools developed by the Cochrane Collaboration. Two authors will independently assess study eligibility, abstract the data, and assess the risk of bias. Random-effects meta-analyses and network meta-analyses will be conducted using the odds ratio for dichotomous outcomes and the mean difference for continuous outcomes. The quality of the evidence for the primary outcome will be assessed using the CINEMA tool.

Ethics and dissemination: Ethical approval not required. We will disseminate findings to clinicians, policy makers, patients and the public.

PROSPERO registration number: CRD42018115508

ARTICLE SUMMARY

Strengths and limitations of this study

- We plan to conduct the first network meta-analysis to assess the relative efficacy and complication rates of treatment methods for cervical pre-invasive and early micro-invasive disease.
- This study will produce comprehensive summaries of the clinical ranking of treatments and will employ methodologies that will allow the use of both randomised and observational data, aiming to utilise all published evidence.
- The results will inform clinicians, patients and clinical guidelines and will allow effective patient counselling at colposcopy clinics.
- We expect to find retrospective observational studies at high risk of recall, selection and publication bias. We will try overcoming this limitation by employing methods that aim to minimise bias.

KEYWORDS

For peer review only

"Cervical Intraepithelial Neoplasia/surgery"[Mesh]
"Cervical Intraepithelial Neoplasia/therapy"[Mesh]
"Treatment Outcome"[Mesh]
"Recurrence"[Mesh]
"Conization/adverse effects"[Mesh]
"Ablation Techniques/adverse effects"[Mesh]

INTRODUCTION

Organised screening programmes in countries such as the UK, have led to a dramatic decrease in the incidence and mortality from cervical cancer, especially when compared with the corresponding statistics for the other major cancers. Over a twenty-year period, from 1993-1995 to 2013-2015, the overall age-standardised incidence rate of cancer in females increased by 16% in the UK[1], whereas the corresponding data for cervical cancer showed a decrease of 24%[2]. Cervical cancer is largely preventable through detection and treatment of the pre-invasive precursor, cervical intra-epithelial neoplasia (CIN)[3]. The local treatment methods are divided into excisional and destructive (ablative) that aim to remove or ablate respectively a cone shaped part of the cervix that contains the 'transformation zone' with the precancerous cells. Although large loop excision of the transformation zone (LLETZ) is the most commonly used methods in the UK[4] given its ease of execution and low cost, the preference of techniques varies across Europe and internationally.

A Cochrane systematic review of RCTs concluded that all local treatment techniques are highly effective in preventing pre-invasive recurrence[5]. However, this review found no evidence of difference in treatment failure rates amongst the treatment techniques. This could be because the RCTs, and the subsequent meta-analysis might have been underpowered to detect differences between the treatments. The largest study recruited only 390 participants[6], while the majority of the rest were much smaller. A more recent, large, population-based study from Sweden[7], which included 150,883 women diagnosed and treated for CIN3 (3,148,222 woman-years), reported a doubled standardized incidence ratio for post-treatment invasive recurrence during the follow-up period of around 4 decades in comparison to the general population, and initiated debates on the impact that less radical treatments may have on the subsequent risk of invasion[8]. The trend towards techniques that remove smaller parts of the cervix can be attributed to the fact that many of these are easy to do, they are of low cost, and can be performed in an outpatient setting. Increased awareness of the impact of the more radical or deeper techniques on the risk of prematurity may have also contributed[9-20].

The impact of different techniques on the risk of pre-invasive and/or invasive recurrence remains therefore unclear. With some advocating the minimum radicality of treatment to prevent treatment-induced reproductive morbidity[10 21], and others raising concerns about the increase in the risk of future invasion[7 8], a definite answer regarding the relative merits and risks among the various treatment strategies is required.

Traditionally, treatment strategies are evaluated via large, expensive trials. Given the possibly comparable and high as well efficacy of most interventions for CIN, it is unlikely that any

For peer review only - http://bmjopen.bmj.com/site/about/guidelines.xhtml

adequately powered RCT assessing the relative efficacy of different treatment techniques will ever be conducted. Such a trial would require thousands of women in order to reach the statistical power needed to detect differences in the pre-invasive and invasive recurrence rates. In summary, there is currently a lack of adequately powered randomised evidence to allow us to compare the various interventions. However, there is a plethora of available observational studies in the field. These studies are a potentially valuable source of evidence, and may act as a complement to the available randomised evidence, allowing us to more accurately assess the comparative effectiveness and safety of the various treatment alternatives. In this paper, we aim to perform a systematic review of both randomised and observational studies in the field, and quantitatively synthesize their findings in meta-analyses.

Systematic reviews and pairwise meta-analyses are widely accepted as a useful tool in comparative effectiveness research, and are commonly used to summarise, critically appraise and synthesise evidence from multiple studies. Investigators aiming to address a research question identify all relevant studies, evaluate their quality, synthesise their findings (meta-analysis) and interpret the provided evidence. Systematic reviews and meta-analyses have played a key role in providing evidence on the efficacy and safety of treatment methods and management strategies in cervical cancer prevention. However, the increased number of management strategies and multiple treatment options requires the use of more advanced evidence-synthesis methods.

Network meta-analysis (NMA) is an extension of pairwise meta-analysis, for the case when multiple treatments are available for the same condition. NMA has been recognised by the National Institute of Clinical Excellence (NICE)[22] and several international Health Technology Assessment (HTA) agencies[23 24] as a methodological tool that has the potential to increase precision in treatment effect estimates but also to infer on the clinical efficacy/ safety between treatments that have never been compared in trials. NMA uses both direct evidence (i.e. coming from studies comparing head-to-head the treatments of interest) and indirect evidence (i.e. coming from studies comparing the treatments of interest via an intermediate common comparator)[25-28], allows the estimation of relative treatment effects between all available interventions, and provides a clinically useful ranking of the different competing treatments. The methodology of NMA has never been used before to assess the comparative efficacy and complications of different treatment techniques used in the management of CIN. Furthermore, novel NMA methodologies will be employed to allow the use of both randomised and observational data.

The aim of this systematic review and NMA is to compare and clinically rank the alternative treatment techniques for CIN based on their efficacy, complications and adverse effects. This NMA forms part of the CIRCLE project (**C**ervical Cancer **I**ncidence, CI**N** **R**ecurrence and **R**eproduction after **L**ocal **E**xcision), which aims to generate a clinically useful raking of alternative options for treatment of CIN according to their efficacy (risk of pre-invasive and invasive recurrence), morbidity and cost-effectiveness.

For peer review only - http://bmjopen.bmj.com/site/about/guidelines.xhtml

METHODS AND ANALYSIS

This protocol is written in accordance with the Preferred Reporting Items for Systematic Review and Meta-Analysis Protocols (PRISMA-P) (see Supplementary File 1)[29]. PROSPERO registration will be updated if we make any amendments to this protocol.

Eligibility criteria of studies

Types of participants

We will include women of all ages treated with local surgical treatment for CIN (or glandular intraepithelial neoplasia) or micro-invasive early cervical cancer (stage IA1). We will only include women with histological diagnosis of CIN.

Types of interventions

The treatment techniques for cervical intraepithelial neoplasia (CIN) are divided into excisional and ablative. The excisional include cold knife conisation (CKC), laser conisation (LC), needle excision of the transformation zone (NETZ), also known as straight wire excision of the transformation zone (SWETZ), large loop excision of the transformation zone (LLETZ), also known as loop electrosurgical excisional procedure (LEEP) and Fischer cone biopsy excisor (FCBE), while the ablative include radical point diathermy (RD), cryotherapy (CT), cold coagulation (CC) and laser ablation (LA). Figure 1 displays a network example of comparisons between studied treatment techniques. When the treatment is not specified, we will group these under wider categories excision (ENS) or ablation (ANS).

Outcome measures

Primary outcome

- High-grade treatment failure rates defined as recurrent or residual histologically-proven CIN2 or worse

Secondary outcomes

- Treatment failure rates defined as histologically-proven CIN1 or worse
- Abnormal cytology (defined as ASCUS or worse) and/or histology CIN1 or worse
- HPV positivity rates
- Involved margins rates (endocervical, ectocervical, or both)
- Peri-operative or post-operative bleeding
- Cervical stenosis

Primary and secondary outcomes were chosen by clinical experts of the team.

Types of studies

We will include RCTs, quasi-RCTs and observational cohort studies comparing rates of treatment failure (recurrent/residual disease) or complications amongst the abovementioned surgical techniques. Single-arm studies not presenting a comparison will be excluded. Studies will be considered regardless of time or language.

For peer review only - http://bmjopen.bmj.com/site/about/guidelines.xhtml

Information sources and search strategy

The Cochrane Gynaecological Cancer Specialised Register, Cochrane Central Register of Controlled Trials (CENTRAL), MEDLINE and EMBASE will be searched for eligible studies by an experienced librarian, as presented in Supplementary File 2. Metaregister, Physicians Data Query, www.controlled-trials.com/rct, www.clinicaltrials.gov and www.cancer.gov/clinicaltrials will be searched for ongoing studies. ZETOC (http://zetoc.mimas.ac.uk) and WorldCat Dissertations will be searched for conference proceedings and theses, respectively. References of the retrieved articles and meta-analyses will be hand-searched, the "related articles" feature in MEDLINE will be employed and experts in the field will be contacted in an attempt to identify further reports of studies. Corresponding authors will be contacted for any relevant ongoing trials and unpublished data.

We will include both published and unpublished data and there will be no time, place or language restriction.

Study selection

Two team members will independently screen titles and abstracts of citations at level 1, using the reference management software Zotero. At level 2, the full text of all potentially eligible articles will be assessed using the same inclusion criteria. Disagreements will be resolved through discussion with a third review author.

Data collection

Data from the included studies will be abstracted at level 3 by two reviewers independently using an a priori developed data collection form in Excel. The following data will be abstracted from the included studies: study characteristics, including author, publication year study design, inclusion/exclusion criteria, and intervention details, participant characteristics, including age, CIN grade and smoking, and dropout rates, and outcome characteristics. In RCTs, we will prefer arm-level data (number of events and sample size per intervention arm for dichotomous data, and mean and standard deviation (SD) per intervention arm for continuous data), but if these are missing, the study-level data will be used in the analysis, e.g. reported odds ratios (ORs) for dichotomous outcomes and mean differences (MDs) for continuous outcomes, along with a measure of uncertainly (e.g., confidence interval [CI]). For continuous outcome data not reported as means and standard deviations, we will first contact the corresponding study authors for further information, but if no additional data are provided, we will perform imputation methods to derive approximate effect measures[30 31]. When an eligible study is observational, we will prefer adjusted treatment effect estimates accounting for the impact of potential confounders, but if these are missing, the unadjusted estimated treatment effects will be abstracted with a corresponding uncertainty measure (e.g. CI). Disagreements will be resolved through consensus or the involvement of a third reviewer.

Risk of bias assessment

RCTs will be assessed for quality and risk of bias using the Cochrane risk of bias tool[32] in the following domains: randomisation process, deviations from the intended interventions,

For peer review only - http://bmjopen.bmj.com/site/about/guidelines.xhtml

missing outcome data, measurement of the outcome and selection of the reported result. The risk of bias in each domain, as well as the overall risk of bias, will be rated as "low risk", "some concerns", or "high risk", after answering the signalling questions of each domain with "Yes", "Potentially Yes", "Potentially No" or "No". Non-randomised studies (NRS) will be assessed using the ROBINS-I tool[33] with potential confounding factors: grade of treated CIN, age and smoking. The following domains will be assessed for NRS: confounding, selection of participants into the study, classification of interventions, deviations from intended interventions, missing data, measurement of outcomes and selection of the reported results. The risk of bias in each domain, as well as the overall risk of bias, will be rated as "low", "moderate", "serious", or "critical", after answering the signalling questions of each domain with "Yes", "Potentially Yes", "Potentially No" or "No". Pairs of team members will independently assess the methodological quality and risk of bias of the eligible studies. Conflicts will be resolved through discussion or with a third investigator. When inadequate information is available from the studies to rate a risk of bias item, we will contact the corresponding study authors for clarification.

Statistical synthesis

Characteristics of included studies and Network

For each outcome, we will produce a network plot (see for example Figure 1) of the available evidence, as well as descriptive statistics, including comparison type, publication year, study design, outcome data, and potential effect modifiers (e.g., age).

Pairwise Meta-analyses

A random-effects meta-analysis will be conducted for each pairwise comparison in each outcome using the inverse variance model and the the Hartung-Knapp-Sidik-Jonkman method to estimate each summary treatment effect and its 95% CI[34-36]. The between-study variance will be estimated with the restricted maximum likelihood estimator, whereas its 95% CI with the Q-profile approach[34 37 38]. We will also use the I^2 statistic along a 95% CI[39 40] to evaluate between-study heterogeneity. For continuous outcomes we will report the summary MDs, whereas for dichotomous outcomes we will use the summary ORs, along with a 95% CI. The *metafor* package[41] in R[42] will be used for all meta-analyses.

Network meta-analyses (NMA)

Data synthesis

A random-effects NMA will be conducted, since we anticipate methodological and clinical between-study heterogeneity. We will assume common between-study variance parameter across treatment comparisons in the network, so that comparisons informed by a single study can borrow strength from the remaining network [43 44]. This assumption is clinically reasonable because all treatments included in the network of trials are of the same nature. The between-study variance will be estimated with the DerSimonian and Laird method of moments approach[45]. We will employ the design-adjusted" and a three-level hierarchical NMA models as described in Efthimiou et al[46] by incorporating all the available information, both RCTs and NRS, in a single NMA model. We will explore the impact of assigning

For peer review only - http://bmjopen.bmj.com/site/about/guidelines.xhtml

different levels of credibility and subsequently down-weighting the NRS according to experts' opinion and the results of the ROBINS-I tool in several sensitivity analyses.

Similar to the pairwise meta-analysis, for continuous outcomes we will report the estimated MDs, whereas for dichotomous outcomes we will use the estimated ORs, with a 95% CI. Along the 95% CI for the summary effect size, we will report 95% prediction intervals to observe the interval within which the effect estimate is expected to lie should another trial become available[47]. To rank the efficacy for each intervention, we will calculate the ranking probabilities for all treatments, the surface under the cumulative ranking curve (SUCRA) or P-scores, and the mean ranks[48 49]. A rank-heat plot will be used to depict the SUCRA values or P-scores across all outcomes[50]. We will apply all NMA models in R[42] using the *netmeta* package[51] and *rjags*[52] package..

Assessment of the transitivity assumption
One of the prerequisite assumptions in NMA is the transitivity assumption, under which the effect modifiers have a similar distribution across treatment comparisons in a network[27 53 54]. Potential effect modifiers expected to influence the estimated treatment effects include year of study, method of ascertainment of exposure/outcome (hospital records, registries or interviews/questionnaires), age, smoking and grade of CIN. For each pairwise comparison with available direct evidence we will summarize these characteristics and will visually inspect the similarity of the identified studies. We will also investigate the inclusion and exclusion criteria of all studies, to make sure that patients, treatments and outcomes in the studies are sufficiently similar in all aspects that might modify relative treatment effects.

Assessment of statistical inconsistency
Consistency in a network of trials will be evaluated both locally and globally. We will first assess the consistency assumption locally by separating the direct from the indirect evidence for every comparison in a network to make judgements about their statistical differences, using the back-calculation method[55]. Then we will assess consistency in each network globally using the design-by-treatment interaction model[56]. We will conceptually explore for potential intransitivity in every network even in the absence of evidence for inconsistency, since the inconsistency tests have low power to detect true inconsistency[57 58]. If no substantial inconsistency is identified in the network of RCTs, we will then evaluate the agreement between RCTs and NRS using the same local and global approaches. Both local (back-calculation method) and global (design-by-treatment interaction model) assessments will be performed under the random-effects model in R[42] using the *netmeta* package[51].

In the NMA including both RCTs and NRS, we will assess for differences between the different study designs[46]. For each treatment comparison we will summarize evidence by up to four different types: direct randomised, indirect randomised, direct non-randomised and indirect non-randomised. If important discrepancies between these types are found, these will be investigated to confirm that the transitivity assumption holds (e.g. when randomised and non-randomised evidence are very different in terms of populations, interventions, etc., the

For peer review only - http://bmjopen.bmj.com/site/about/guidelines.xhtml

transitivity assumption may be violated). If disagreement occurs for a certain characteristic, this will be explored through a network meta-regression model[26].

Exploring heterogeneity and inconsistency: subgroup analyses and meta-regression
The between-study heterogeneity will be explored by comparing the estimated between-study variance with the empirical distribution derived by Rhodes et al for continuous data[59] and the one derived by Turner et al for dichotomous data[60]. We will also compare 95% CIs with the 95% prediction intervals to infer on the magnitude of the between-study variance.

If at least 10 studies are available, the following potential sources of heterogeneity and/or inconsistency will be explored for the primary outcome using subgroup or meta-regression analyses: year of study, method of ascertainment of exposure/outcome (hospital records, registries or interviews/questionnaires), age, smoking and grade of CIN.

Reporting bias and small study effects
We will assess small-study effects by visually exploring the funnel-plot for each treatment, and the comparison adjusted funnel plot[61] when at least 10 studies are available. We will also conduct a network meta-regression using the study variance as a covariate[62 63].

Assessment of the credibility of the evidence
For the primary outcome, two team members will determine the degree of confidence in the estimated NMA results using CINEMA[64] and the relevant online tool (http://cinema.ispm.ch/). The six CINEMA domains, within-study bias (i.e., risk of bias in the included studies), across-study bias (i.e., publication and reporting bias), indirectness, imprecision, heterogeneity and incoherence (i.e., differences between direct and indirect evidence)[64], will first be rated as high quality, and then they will be downgraded if judged appropriate to moderate, low, or very low quality.

ETHICS AND DISSEMINATION
We do not require ethical approval for this review. We aim to disseminate the results to clinicians, academic researchers, health agencies and decision makers, to patients and the public. We will publish the results in high impact open access journals and disseminate findings though presentations at medical conferences. The data will become available in public repositories. We will develop information sheets and briefings, highlighting the key findings and circulate newsletters. We will work closed with the Jo's Trust, charity in cervical cancer that frequently organises events to educate the public and also engage the media with interviews. We circulate findings in the Imperial College webpage and will circulate newsletters.

Figure 1. Network of possible pairwise comparisons between eligible treatment methods
Abbreviations: **FCBE**=Fischer cone biopsy excision; **LLETZ**= large loop excision of the transformation zone, also known as **LEEP**=loop electrosurgical excisional procedure; **NETZ**=needle excision of the transformation, also known as **SWETZ**=straight wire excision

For peer review only - http://bmjopen.bmj.com/site/about/guidelines.xhtml

of the transformation zone; **LC**=laser conisation; **CKC**=cold knife conisation; **LA**=laser ablation; **CC**=cold coagulation; **CT**=cryotherapy; **RD**=radical point diathermy

REFERENCES

1. CancerResearchUK. Cancer incidence for all cancers combined 2018 [Available from: https://www.cancerresearchuk.org/health-professional/cancer-statistics/incidence/all-cancers-combined.
2. CancerResearchUK. Cervical cancer incidence statistics 2018 [Available from: https://www.cancerresearchuk.org/health-professional/cancer-statistics/statistics-by-cancer-type/cervical-cancer/incidence.
3. Scarinci IC, Garcia FA, Kobetz E, et al. Cervical cancer prevention: new tools and old barriers. *Cancer* 2010;116(11):2531-42. doi: 10.1002/cncr.25065 [published Online First: 2010/03/24]
4. NHS. Colposcopy: Treatments 2017 [Available from: https://www.nhs.uk/conditions/colposcopy/treatment/.
5. Martin-Hirsch PP, Paraskevaidis E, Bryant A, et al. Surgery for cervical intraepithelial neoplasia. *Cochrane Database Syst Rev* 2013(12):Cd001318. doi: 10.1002/14651858.CD001318.pub3 [published Online First: 2013/12/05]
6. Mitchell MF, Tortolero-Luna G, Cook E, et al. A randomized clinical trial of cryotherapy, laser vaporization, and loop electrosurgical excision for treatment of squamous intraepithelial lesions of the cervix. *Obstetrics and gynecology CNO - CN-00156163* 1998;92(5 XMAN - MEDLINE 1999008792 XMO - SU XSR - SR-GYNAECA):737-44.
7. Strander B, Hällgren J, Sparén P. Effect of ageing on cervical or vaginal cancer in Swedish women previously treated for cervical intraepithelial neoplasia grade 3: population based cohort study of long term incidence and mortality. *BMJ* 2014;348:f7361. [published Online First: 2014/01/14]
8. Arbyn M, Kyrgiou M, Gondry J, et al. Long term outcomes for women treated for cervical precancer. *Bmj* 2014;348:f7700. doi: 10.1136/bmj.f7700 [published Online First: 2014/01/16]
9. Kyrgiou M, Koliopoulos G, Martin-Hirsch P, et al. Obstetric outcomes after conservative treatment for intraepithelial or early invasive cervical lesions: systematic review and meta-analysis. *Lancet* 2006;367(9509):489-98. doi: 10.1016/s0140-6736(06)68181-6 [published Online First: 2006/02/14]
10. Arbyn M, Kyrgiou M, Simoens C, et al. Perinatal mortality and other severe adverse pregnancy outcomes associated with treatment of cervical intraepithelial neoplasia: meta-analysis. *Bmj* 2008;337:a1284. doi: 10.1136/bmj.a1284 [published Online First: 2008/09/20]
11. Kyrgiou M, Mitra A, Arbyn M, et al. Fertility and early pregnancy outcomes after treatment for cervical intraepithelial neoplasia: systematic review and meta-analysis. *Bmj* 2014;349:g6192. doi: 10.1136/bmj.g6192 [published Online First: 2014/10/30]
12. Kyrgiou M, Mitra A, Arbyn M, et al. Fertility and early pregnancy outcomes after conservative treatment for cervical intraepithelial neoplasia. *Cochrane Database Syst*

For peer review only - http://bmjopen.bmj.com/site/about/guidelines.xhtml

Rev 2015(9):Cd008478. doi: 10.1002/14651858.CD008478.pub2 [published Online First: 2015/09/30]
13. Kyrgiou M, Athanasiou A, Paraskevaidi M, et al. Adverse obstetric outcomes after local treatment for cervical preinvasive and early invasive disease according to cone depth: systematic review and meta-analysis. *Bmj* 2016;354:i3633. doi: 10.1136/bmj.i3633 [published Online First: 2016/07/30]
14. Kyrgiou M, Athanasiou A, Kalliala IEJ, et al. Obstetric outcomes after conservative treatment for cervical intraepithelial lesions and early invasive disease. *Cochrane Database Syst Rev* 2017;11:Cd012847. doi: 10.1002/14651858.Cd012847 [published Online First: 2017/11/03]
15. Kyrgiou M, Arbyn M, Martin-Hirsch P, et al. Increased risk of preterm birth after treatment for CIN. *Bmj* 2012;345:e5847. doi: 10.1136/bmj.e5847 [published Online First: 2012/09/07]
16. Castanon A, Landy R, Brocklehurst P, et al. Risk of preterm delivery with increasing depth of excision for cervical intraepithelial neoplasia in England: nested case-control study. *BMJ* 2014;349:g6223. [published Online First: 2014/11/05]
17. Noehr B, Jensen A, Frederiksen K, et al. Depth of cervical cone removed by loop electrosurgical excision procedure and subsequent risk of spontaneous preterm delivery. *Obstet Gynecol* 2009;114(6):1232-8. doi: 10.1097/AOG.0b013e3181bf1ef2 [published Online First: 2009/11/26]
18. Khalid S, Dimitriou E, Conroy R, et al. The thickness and volume of LLETZ specimens can predict the relative risk of pregnancy-related morbidity. *Bjog* 2012;119(6):685-91. doi: 10.1111/j.1471-0528.2011.03252.x [published Online First: 2012/02/15]
19. Founta C, Arbyn M, Valasoulis G, et al. Proportion of excision and cervical healing after large loop excision of the transformation zone for cervical intraepithelial neoplasia. *Bjog* 2010;117(12):1468-74. doi: 10.1111/j.1471-0528.2010.02709.x [published Online First: 2010/09/16]
20. Kyrgiou M, Valasoulis G, Stasinou SM, et al. Proportion of cervical excision for cervical intraepithelial neoplasia as a predictor of pregnancy outcomes. *Int J Gynaecol Obstet* 2015;128(2):141-7. doi: 10.1016/j.ijgo.2014.07.038 [published Online First: 2014/12/03]
21. Paraskevaidis E, Kyrgiou M, Martin-Hirsch P. Have we dismissed ablative treatment too soon in colposcopy practice? *Bjog* 2007;114(1):3-4. doi: 10.1111/j.1471-0528.2006.01178.x [published Online First: 2007/01/20]
22. NICE. Developing NICE guidelines: the manual. Process and methods (PMG20). Reviewing research evidence 2017 [Available from: https://www.nice.org.uk/process/pmg20/chapter/reviewing-research-evidence.
23. Caldwell DM, Dias S, Welton NJ. Extending Treatment Networks in Health Technology Assessment: How Far Should We Go? *Value Health* 2015;18(5):673-81. doi: 10.1016/j.jval.2015.03.1792 [published Online First: 2015/08/25]
24. CADTH. Network Meta-Analysis 2015 [Available from: https://www.cadth.ca/network-meta-analysis.

For peer review only - http://bmjopen.bmj.com/site/about/guidelines.xhtml

25. Caldwell DM, Ades AE, Higgins JP. Simultaneous comparison of multiple treatments: combining direct and indirect evidence. *BMJ* 2005;331(7521):897-900. doi: 10.1136/bmj.331.7521.897
26. Salanti G, Marinho V, Higgins JP. A case study of multiple-treatments meta-analysis demonstrates that covariates should be considered. *J Clin Epidemiol* 2009;62(8):857-64. doi: 10.1016/j.jclinepi.2008.10.001 [published Online First: 2009/01/20]
27. Cipriani A, Higgins JP, Geddes JR, et al. Conceptual and technical challenges in network meta-analysis. *Ann Intern Med* 2013;159(2):130-7. doi: 10.7326/0003-4819-159-2-201307160-00008
28. Efthimiou O, Debray TP, van Valkenhoef G, et al. GetReal in network meta-analysis: a review of the methodology. *Res Synth Methods* 2016;7(3):236-63. doi: 10.1002/jrsm.1195 [published Online First: 2016/01/13]
29. Moher D, Shamseer L, Clarke M, et al. Preferred reporting items for systematic review and meta-analysis protocols (PRISMA-P) 2015 statement. *Syst Rev* 2015;4:1. doi: 10.1186/2046-4053-4-1 [published Online First: 2015/01/03]
30. Furukawa TA, Barbui C, Cipriani A, et al. Imputing missing standard deviations in meta-analyses can provide accurate results. *J Clin Epidemiol* 2006;59(1):7-10. doi: 10.1016/j.jclinepi.2005.06.006 [published Online First: 2005/12/20]
31. Higgins JP, Altman DG, Gotzsche PC, et al. The Cochrane Collaboration's tool for assessing risk of bias in randomised trials. *BMJ* 2011;343:d5928. doi: 10.1136/bmj.d5928 [published Online First: 2011/10/20]
32. Higgins JPT, Sterne JAC, Savović J, et al. A revised tool for assessing risk of bias in randomized trials. In: Chandler J, McKenzie J, Boutron I, et al., eds.: Cochrane Methods. Cochrane Database of Systematic Reviews 2016.
33. Sterne JA, Hernan MA, Reeves BC, et al. ROBINS-I: a tool for assessing risk of bias in non-randomised studies of interventions. *BMJ* 2016;355:i4919. doi: 10.1136/bmj.i4919 [published Online First: 2016/10/14]
34. Veroniki AA, Jackson D, Viechtbauer W, et al. Methods to estimate the between-study variance and its uncertainty in meta-analysis. *Res Synth Methods* 2016;7(1):55-79. doi: 10.1002/jrsm.1164 [published Online First: 2015/09/04]
35. Hartung J, Knapp G. A refined method for the meta-analysis of controlled clinical trials with binary outcome. *Stat Med* 2001;20(24):3875-89. [published Online First: 2002/01/10]
36. Sidik K, Jonkman JN. A simple confidence interval for meta-analysis. *Stat Med* 2002;21(21):3153-9. doi: 10.1002/sim.1262 [published Online First: 2002/10/11]
37. Langan D, Higgins JPT, Jackson D, et al. A comparison of heterogeneity variance estimators in simulated random-effects meta-analyses. *Res Synth Methods* 2018 doi: 10.1002/jrsm.1316 [published Online First: 2018/08/02]
38. Viechtbauer W. Confidence intervals for the amount of heterogeneity in meta-analysis. *Stat Med* 2007;26(1):37-52. doi: 10.1002/sim.2514 [published Online First: 2006/02/08]
39. Higgins JP, Thompson SG, Deeks JJ, et al. Measuring inconsistency in meta-analyses. *BMJ* 2003;327(7414):557-60. doi: 10.1136/bmj.327.7414.557 [published Online First: 2003/09/06]

For peer review only - http://bmjopen.bmj.com/site/about/guidelines.xhtml

40. Veroniki AA, Jackson D, Viechtbauer W, et al. Recommendations for quantifying the uncertainty in the summary intervention effect and estimating the between-study heterogeneity variance in random-effects meta-analysis. In: Chandler J, McKenzie J, Boutron I, et al., eds.: Cochrane Methods. Cochrane Database of Systematic Reviews 2015.
41. Viechtbauer W. Conducting Meta-Analyses in R with the metafor Package. 2010;36(3):48.
42. RDevelopmentCoreTeam. R: A language and environment for statistical computing. Vienna, Austria: R Foundation for Statistical Computing, 2013.
43. Salanti G, Higgins JP, Ades AE, et al. Evaluation of networks of randomized trials. *Stat Methods Med Res* 2008;17(3):279-301. doi: 10.1177/0962280207080643 [published Online First: 2007/10/09]
44. Higgins JP, Whitehead A. Borrowing strength from external trials in a meta-analysis. *Stat Med* 1996;15(24):2733-49. doi: 10.1002/(SICI)1097-0258(19961230)15:24<2733::AID-SIM562>3.0.CO;2-0
45. Jackson D, White IR, Riley RD. Quantifying the impact of between-study heterogeneity in multivariate meta-analyses. *Stat Med* 2012;31(29):3805-20. doi: 10.1002/sim.5453 [published Online First: 2012/07/06]
46. Efthimiou O, Mavridis D, Debray TP, et al. Combining randomized and non-randomized evidence in network meta-analysis. *Stat Med* 2017;36(8):1210-26. doi: 10.1002/sim.7223 [published Online First: 2017/01/14]
47. Riley RD, Higgins JP, Deeks JJ. Interpretation of random effects meta-analyses. *BMJ* 2011;342:d549. doi: 10.1136/bmj.d549 [published Online First: 2011/02/12]
48. Salanti G, Ades AE, Ioannidis JP. Graphical methods and numerical summaries for presenting results from multiple-treatment meta-analysis: an overview and tutorial. *J Clin Epidemiol* 2011;64(2):163-71. doi: 10.1016/j.jclinepi.2010.03.016 [published Online First: 2010/08/05]
49. Rucker G, Schwarzer G. Ranking treatments in frequentist network meta-analysis works without resampling methods. *BMC medical research methodology* 2015;15:58. doi: 10.1186/s12874-015-0060-8 [published Online First: 2015/08/01]
50. Veroniki AA, Straus SE, Fyraridis A, et al. The rank-heat plot is a novel way to present the results from a network meta-analysis including multiple outcomes. *J Clin Epidemiol* 2016;76:193-9. doi: 10.1016/j.jclinepi.2016.02.016 [published Online First: 2016/03/05]
51. Rücker G, Schwarzer G, Krahn U, et al. netmeta: Network Meta-Analysis using Frequentist Methods. R package version 0.9-8. 2018 [Available from: https://CRAN.R-project.org/package=netmeta.
52. Plummer M. rjags: Bayesian Graphical Models using MCMC. R package version 4-8 2018 [Available from: https://CRAN.R-project.org/package=rjags.
53. Salanti G. Indirect and mixed-treatment comparison, network, or multiple-treatments meta-analysis: many names, many benefits, many concerns for the next generation evidence synthesis tool. *Res Synth Methods* 2012;3(2):80-97. doi: 10.1002/jrsm.1037 [published Online First: 2012/06/11]

For peer review only - http://bmjopen.bmj.com/site/about/guidelines.xhtml

54. Jansen JP, Naci H. Is network meta-analysis as valid as standard pairwise meta-analysis? It all depends on the distribution of effect modifiers. *BMC Med* 2013;11:159. doi: 10.1186/1741-7015-11-159 [published Online First: 2013/07/04]
55. Konig J, Krahn U, Binder H. Visualizing the flow of evidence in network meta-analysis and characterizing mixed treatment comparisons. *Stat Med* 2013;32(30):5414-29. doi: 10.1002/sim.6001 [published Online First: 2013/10/15]
56. White IR, Barrett JK, Jackson D, et al. Consistency and inconsistency in network meta-analysis: model estimation using multivariate meta-regression. *Res Synth Methods* 2012;3(2):111-25. doi: 10.1002/jrsm.1045
57. Veroniki AA, Mavridis D, Higgins JP, et al. Characteristics of a loop of evidence that affect detection and estimation of inconsistency: a simulation study. *BMC medical research methodology* 2014;14:106. doi: 10.1186/1471-2288-14-106 [published Online First: 2014/09/23]
58. Song F, Clark A, Bachmann MO, et al. Simulation evaluation of statistical properties of methods for indirect and mixed treatment comparisons. *BMC medical research methodology* 2012;12:138. doi: 10.1186/1471-2288-12-138 [published Online First: 2012/09/14]
59. Rhodes KM, Turner RM, Higgins JP. Predictive distributions were developed for the extent of heterogeneity in meta-analyses of continuous outcome data. *J Clin Epidemiol* 2015;68(1):52-60. doi: 10.1016/j.jclinepi.2014.08.012 [published Online First: 2014/10/12]
60. Turner RM, Davey J, Clarke MJ, et al. Predicting the extent of heterogeneity in meta-analysis, using empirical data from the Cochrane Database of Systematic Reviews. *Int J Epidemiol* 2012;41(3):818-27. doi: 10.1093/ije/dys041 [published Online First: 2012/03/31]
61. Chaimani A, Higgins JP, Mavridis D, et al. Graphical tools for network meta-analysis in STATA. *PLoS One* 2013;8(10):e76654. doi: 10.1371/journal.pone.0076654 [published Online First: 2013/10/08]
62. Chaimani A, Salanti G. Using network meta-analysis to evaluate the existence of small-study effects in a network of interventions. *Res Synth Methods* 2012;3(2):161-76. doi: 10.1002/jrsm.57 [published Online First: 2012/06/01]
63. Mavridis D, Efthimiou O, Leucht S, et al. Publication bias and small-study effects magnified effectiveness of antipsychotics but their relative ranking remained invariant. *J Clin Epidemiol* 2016;69:161-9. doi: 10.1016/j.jclinepi.2015.05.027 [published Online First: 2015/07/27]
64. Salanti G, Del Giovane C, Chaimani A, et al. Evaluating the quality of evidence from a network meta-analysis. *PLoS One* 2014;9(7):e99682. doi: 10.1371/journal.pone.0099682 [published Online First: 2014/07/06]

STATEMENTS

Declaration

The corresponding author had full access to all the data in the study and the final responsibility for the decision to submit for publication.

For peer review only

The senior author MK (the manuscript's guarantor) affirms that the manuscript is an honest, accurate, and transparent account of the study being reported and that no important aspects of the study have been omitted.

Author statement

The study was conceived and designed by MK, GS and EP. The protocol was drafted by AA, MK, AAV, OE, IK, GS and was revised critically for important intellectual content by all authors (AA, AAV, OE, IK, HN, SL, MP, PMH, PB, EP, GS, MK).

Acknowledgements

All contributors are listed as authors.

Funding statement

This work is supported by National Institute for Health Research (NIHR) Research for Patient Benefits (P67307)(MK). It is also supported by the British Society of Colposcopy Cervical Pathology Jordan/Singer Award (P47773) and the Imperial College Healthcare Charity (P47907)(MK). AAV is funded by the European Union's Horizon 2020 (No 754936). None of the funders have any influence on the study design; in the collection, analysis, and interpretation of data; in the writing of the report; and in the decision to submit the article for publication.

Competing interests statement

We have read and understood BMJ policy on declaration of interests and declare that we have no conflict of interests. All authors have completed the ICMJE uniform disclosure form at www.icmje.org/coi_disclosure.pdf and declare: no support from any organisation for the submitted work; no financial relationships with any organisations that might have an interest in the submitted work in the previous three years; no other relationships or activities that could appear to have influenced the submitted work**.**

Dr Kyrgiou has received travel and conference expenses, honoraria and consultancy fees for commercial companies (Inovio, MSD etc); these activities are not related to the project.

Data sharing

No additional data available.

Copyright statement

"The Corresponding Author has the right to grant on behalf of all authors and does grant on behalf of all authors, a worldwide licence to the Publishers and its licensees in perpetuity, in all forms, formats and media (whether known now or created in the future), to i) publish, reproduce, distribute, display and store the Contribution, ii) translate the Contribution into other languages, create adaptations, reprints, include within collections and create summaries, extracts and/or, abstracts of the Contribution, iii) create any other derivative work(s) based on the Contribution, iv) to exploit all subsidiary rights in the Contribution, v) the inclusion of electronic links from the Contribution to third party material where-ever it may be located; and, vi) licence any third party to do any or all of the above."

For peer review only - http://bmjopen.bmj.com/site/about/guidelines.xhtml

For peer review only

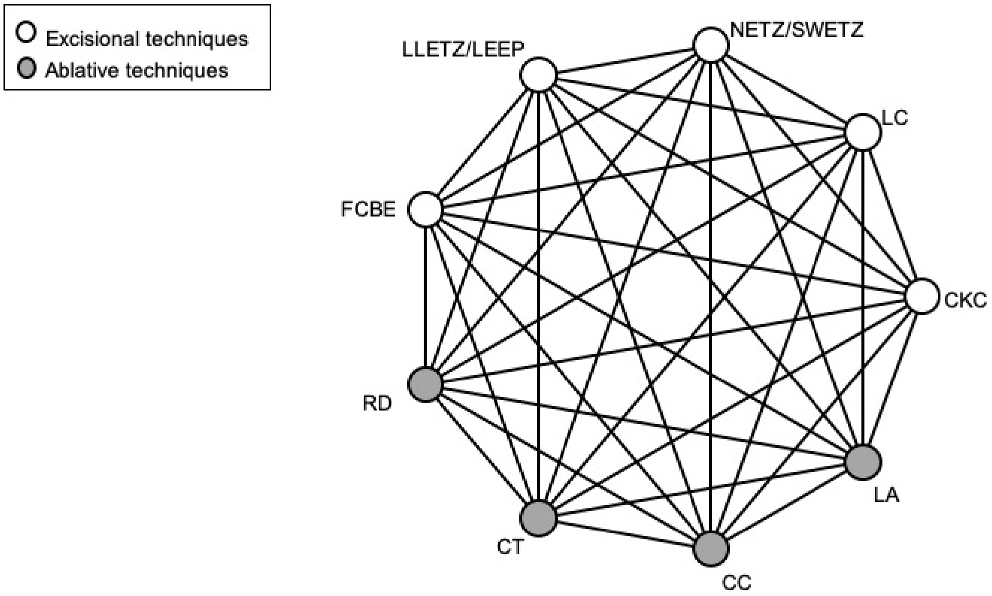

Network of possible pairwise comparisons between eligible treatment methods

235x142mm (72 x 72 DPI)

Supplementary File 1: Reporting checklist for protocol of a systematic review

Based on the PRISMA-P guidelines.

		Reporting Item	Page Number
Identification	#1a	Identify the report as a protocol of a systematic review	1
Update	#1b	If the protocol is for an update of a previous systematic review, identify as such	N/A (no previous NMA)
	#2	If registered, provide the name of the registry (such as PROSPERO) and registration number	2
Contact	#3a	Provide name, institutional affiliation, e-mail address of all protocol authors; provide physical mailing address of corresponding author	1
Contribution	#3b	Describe contributions of protocol authors and identify the guarantor of the review	14
	#4	If the protocol represents an amendment of a previously completed or published protocol, identify as such and list changes; otherwise, state plan for documenting important protocol amendments	4
Sources	#5a	Indicate sources of financial or other support for the review	15
Sponsor	#5b	Provide name for the review funder and / or sponsor	15
Role of sponsor or funder	#5c	Describe roles of funder(s), sponsor(s), and / or institution(s), if any, in developing the protocol	15
Rationale	#6	Describe the rationale for the review in the context of what is already known	3-4
Objectives	#7	Provide an explicit statement of the question(s) the review will address with reference to participants, interventions, comparators, and outcomes (PICO)	4
Eligibility criteria	#8	Specify the study characteristics (such as PICO, study design, setting, time frame) and report characteristics (such as years considered, language, publication status) to be used as criteria for eligibility for the review	5

For peer review only

Information sources	#9	Describe all intended information sources (such as electronic databases, contact with study authors, trial registers or other grey literature sources) with planned dates of coverage	5-6
Search strategy	#10	Present draft of search strategy to be used for at least one electronic database, including planned limits, such that it could be repeated	5; Supplementary File 2
Study records - data management	#11a	Describe the mechanism(s) that will be used to manage records and data throughout the review	6
Study records - selection process	#11b	State the process that will be used for selecting studies (such as two independent reviewers) through each phase of the review (that is, screening, eligibility and inclusion in meta-analysis)	6
Study records - data collection process	#11c	Describe planned method of extracting data from reports (such as piloting forms, done independently, in duplicate), any processes for obtaining and confirming data from investigators	6
Data items	#12	List and define all variables for which data will be sought (such as PICO items, funding sources), any pre-planned data assumptions and simplifications	6
Outcomes and prioritization	#13	List and define all outcomes for which data will be sought, including prioritization of main and additional outcomes, with rationale	5
Risk of bias in individual studies	#14	Describe anticipated methods for assessing risk of bias of individual studies, including whether this will be done at the outcome or study level, or both; state how this information will be used in data synthesis	6-7
Data synthesis	#15a	Describe criteria under which study data will be quantitatively synthesised	7
	#15b	If data are appropriate for quantitative synthesis, describe planned summary measures, methods of handling data and methods of combining data from studies, including any planned exploration of consistency (such as I2, Kendall's τ)	7-8
	#15c	Describe any proposed additional analyses (such as sensitivity or subgroup analyses, meta-regression)	8-9
	#15d	If quantitative synthesis is not appropriate, describe the type of	N/A

For peer review only

		summary planned	
Meta-bias(es)	#16	Specify any planned assessment of meta-bias(es) (such as publication bias across studies, selective reporting within studies)	9
Confidence in cumulative evidence	#17	Describe how the strength of the body of evidence will be assessed (such as GRADE)	9

The PRISMA-P checklist is distributed under the terms of the Creative Commons Attribution License CC-BY 4.0. This checklist can be completed online using https://www.goodreports.org/, a tool made by the EQUATOR Network in collaboration with Penelope.ai

For peer review only

Supplementary File 2: Search algorithms

Medline Ovid RCT only

1 exp Cervical Intraepithelial Neoplasia/
2 CIN.mp.
3 (cervi* and (intraepithel* or epithel*)).mp.
4 (cervi* and dysplasia).mp.
5 (cervi* and carcinoma in situ).mp.
6 (cervi* and cancer in situ).mp.
7 (cervi* and (precancer* or pre-cancer*)).mp.
8 1 or 2 or 3 or 4 or 5 or 6 or 7
9 surgery.fs.
10 exp Gynecologic Surgical Procedures/
11 (surg* or ablat* or excis* or cryotherapy or laser* or cone or conisation or biopsy or transformation zone or LLETZ or LEEP).mp.
12 9 or 10 or 11
13 8 and 12
14 randomized controlled trial.pt.
15 controlled clinical trial.pt.
16 randomized.ab.
17 placebo.ab.
18 clinical trials as topic.sh.
19 randomly.ab.
20 trial.ti.
21 14 or 15 or 16 or 17 or 18 or 19 or 20
22 13 and 21

Medline Ovid NON RCT only

1. exp Cervical Intraepithelial Neoplasia/
2. CIN.mp. [mp=title, abstract, original title, name of substance word, subject heading word, keyword heading word, protocol supplementary concept word, rare disease supplementary concept word, unique identifier, synonyms]
3. (cervi* and (intraepithel* or epithel*)).mp. [mp=title, abstract, original title, name of substance word, subject heading word, keyword heading word, protocol supplementary concept word, rare disease supplementary concept word, unique identifier, synonyms]
4. (cervi* and dysplasia).mp. [mp=title, abstract, original title, name of substance word, subject heading word, keyword heading word, protocol supplementary concept word, rare disease supplementary concept word, unique identifier, synonyms]

For peer review only

5. (cervi* and carcinoma in situ).mp. [mp=title, abstract, original title, name of substance word, subject heading word, keyword heading word, protocol supplementary concept word, rare disease supplementary concept word, unique identifier, synonyms]
6. (cervi* and cancer in situ).mp. [mp=title, abstract, original title, name of substance word, subject heading word, keyword heading word, protocol supplementary concept word, rare disease supplementary concept word, unique identifier, synonyms]
7. (cervi* and (precancer* or pre-cancer*)).mp. [mp=title, abstract, original title, name of substance word, subject heading word, keyword heading word, protocol supplementary concept word, rare disease supplementary concept word, unique identifier, synonyms]
8. 1 or 2 or 3 or 4 or 5 or 6 or 7
9. surgery.fs.
10. exp Gynecologic Surgical Procedures/
11. (surg* or ablat* or excis* or cryotherapy or laser* or cone or conisation or biopsy or transformation zone or LLETZ or LEEP).mp. [mp=title, abstract, original title, name of substance word, subject heading word, keyword heading word, protocol supplementary concept word, rare disease supplementary concept word, unique identifier, synonyms]
12. 9 or 10 or 11
13. 8 and 12
14. randomized controlled trial.pt.
15. controlled clinical trial.pt.
16. randomized.ab.
17. placebo.ab.
18. clinical trials as topic.sh.
19. randomly.ab.
20. trial.ti.
21. groups.ab.
22. exp cohort studies/
23. exp case-control studies/
24. (cohort* or prospective* or retrospective* or (case* and (control* or series))).mp.
25. 14 or 15 or 16 or 17 or 18 or 19 or 20 or 21 or 22 or 23 or 24
26. (animals not (humans and animals)).sh.
27. 25 not 26
28. 13 and 27

Embase Ovid RCT only

1 exp Uterine Cervix Carcinoma in Situ/

For peer review only

2 CIN.mp.
3 (cervi* and (intraepithel* or epithel*)).mp.
4 (cervi* and dysplasia).mp.
5 (cervi* and carcinoma in situ).mp.
6 (cervi* and cancer in situ).mp.
7 (cervi* and (precancer* or pre-cancer*)).mp.
8 1 or 2 or 3 or 4 or 5 or 6 or 7
9 su.fs.
10 exp gynecologic surgery/
11 (surg* or ablat* or excis* or cryotherapy or laser* or cone or conisation or biopsy or transformation zone or LLETZ or LEEP).mp.
12 9 or 10 or 11
13 8 and 12
14 crossover procedure/
15 double-blind procedure/
16 randomized controlled trial/
17 single-blind procedure/
18 random*.mp.
19 factorial*.mp.
20 (crossover* or cross over* or cross-over*).mp.
21 placebo*.mp.
22 (double* adj blind*).mp.
23 (singl* adj blind*).mp.
24 assign*.mp.
25 allocat*.mp.
26 volunteer*.mp.
27 14 or 15 or 16 or 17 or 18 or 19 or 20 or 21 or 22 or 23 or 24 or 25 or 26
28 13 and 27

Embase Ovid All Studies

1. exp Uterine Cervix Carcinoma in Situ/
2. CIN.mp. [mp=title, abstract, heading word, drug trade name, original title, device manufacturer, drug manufacturer, device trade name, keyword, floating subheading word]
3. (cervi* and (intraepithel* or epithel*)).mp. [mp=title, abstract, heading word, drug trade name, original title, device manufacturer, drug manufacturer, device trade name, keyword, floating subheading word]

For peer review only

4. (cervi* and dysplasia).mp. [mp=title, abstract, heading word, drug trade name, original title, device manufacturer, drug manufacturer, device trade name, keyword, floating subheading word]
5. (cervi* and carcinoma in situ).mp. [mp=title, abstract, heading word, drug trade name, original title, device manufacturer, drug manufacturer, device trade name, keyword, floating subheading word]
6. (cervi* and cancer in situ).mp. [mp=title, abstract, heading word, drug trade name, original title, device manufacturer, drug manufacturer, device trade name, keyword, floating subheading word]
7. (cervi* and (precancer* or pre-cancer*)).mp. [mp=title, abstract, heading word, drug trade name, original title, device manufacturer, drug manufacturer, device trade name, keyword, floating subheading word]
8. 1 or 2 or 3 or 4 or 5 or 6 or 7
9. su.fs.
10. exp gynecologic surgery/
11. (surg* or ablat* or excis* or cryotherapy or laser* or cone or conisation or biopsy or transformation zone or LLETZ or LEEP).mp.
12. 9 or 10 or 11
13. 8 and 12
14. exp controlled clinical trial/
15. randomized.ab.
16. randomly.ab.
17. trial.ab.
18. groups.ab.
19. exp cohort analysis/
20. cohort*.mp.
21. exp retrospective study/
22. exp prospective study/
23. (case* and series).mp.
24. 14 or 15 or 16 or 17 or 18 or 19 or 20 or 21 or 22 or 23
25. 13 and 24

CENTRAL
#1 MeSH descriptor Cervical Intraepithelial Neoplasia explode all trees
#2 CIN
#3 cervi* and (intraepithel* or epithel*)
#4 cervi* and dysplasia
#5 cervi* and carcinoma in situ
#6 cervi* and cancer in situ
#7 cervi* and (precancer* or pre-cancer*)
#8 (#1 OR #2 OR #3 OR #4 OR #5 OR #6 OR #7)

For peer review only

#9 Any MeSH descriptor with qualifier(s): [Surgery - SU]
#10 MeSH descriptor: [Gynecologic Surgical Procedures] explode all trees
#11 surg* or ablat* or excis* or cryotherapy or laser* or cone or conisation or biopsy or transformation zone or LLETZ or LEEP
#12 #9 or #10 or #11
#13 #8 and #12

For peer review only

BMJ Open

Comparative efficacy and complication rates after local treatment for cervical intra-epithelial neoplasia and stage 1a1 cervical cancer: protocol for a systematic review and network meta-analysis from the CIRCLE Group

Journal:	*BMJ Open*
Manuscript ID	bmjopen-2018-028008.R1
Article Type:	Protocol
Date Submitted by the Author:	07-Jun-2019
Complete List of Authors:	Athanasiou, Antonios; Institute of Reproductive and Developmental Biology, Department of Surgery & Cancer, Faculty of Medicine, Imperial College London; Imperial College Healthcare NHS Trust<br>Veroniki, Areti Angeliki; Institute of Reproductive and Developmental Biology, Department of Surgery & Cancer, Faculty of Medicine, Imperial College London; Panepistimio Ioanninon, School of Education, Department of Primary Education<br>Efthimiou, Orestis; University of Bern, Institute of Social and Preventive Medicine (ISPM)<br>Kalliala, Ilkka; Institute of Reproductive and Developmental Biology, Department of Surgery & Cancer, Faculty of Medicine, Imperial College London; University of Helsinki and Helsinki University Hospital, Department of Obstetrics and Gynaecology<br>Naci, Huseyin; London School of Economics, Department of Health Policy<br>Lever, Sarah; Institute of Reproductive and Developmental Biology, Department of Surgery & Cancer, Faculty of Medicine, Imperial College London; Imperial College Healthcare NHS Trust<br>Paraskevaidi, Maria; Institute of Reproductive and Developmental Biology, Department of Surgery & Cancer, Faculty of Medicine, Imperial College London<br>Martin-Hirsch, Pierre; Lancashire Teaching Hospitals NHS Foundation Trust, Department of Gynaecologic Oncology<br>Bennett, Philip; Institute of Reproductive and Developmental Biology, Department of Surgery & Cancer, Faculty of Medicine, Imperial College London; Imperial College Healthcare NHS Trust<br>Paraskevaidis, Evangelos; University of Ioannina and University Hospital of Ioannina, Department of Obstetrics & Gynaecology; Imperial College Healthcare NHS Trust<br>Salanti, Georgia; University of Bern, Institute of Social and Preventive Medicine (ISPM)<br>Kyrgiou, Maria; Institute of Reproductive and Developmental Biology, Department of Surgery & Cancer, Faculty of Medicine, Imperial College London; Imperial College Healthcare NHS Trust
**Primary Subject Heading**:	Obstetrics and gynaecology
Secondary Subject Heading:	Evidence based practice

Keywords:	"Cervical Intraepithelial Neoplasia/surgery"[Mesh], "Cervical Intraepithelial Neoplasia/therapy"[Mesh], "Treatment Outcome"[Mesh], "Recurrence"[Mesh], "Conization/adverse effects"[Mesh], "Ablation Techniques/adverse effects"[Mesh]

SCHOLARONE™
Manuscripts

Comparative efficacy and complication rates after local treatment for cervical intra-epithelial neoplasia and stage 1a1 cervical cancer: protocol for a systematic review and network meta-analysis from the CIRCLE Group

Antonios Athanasiou[1,2], Areti Angeliki Veroniki[1,3], Orestis Efthimiou[4], Ilkka Kalliala[1,5], Huseyin Naci[6], Sarah Lever[1,2], Maria Paraskevaidi[1], Pierre Martin-Hirsch[7], Philip Bennett[1,2], Evangelos Paraskevaidis[2,8], Georgia Salanti[4], Maria Kyrgiou[1,2]

[1]Institute of Reproductive and Developmental Biology, Department of Surgery & Cancer, Faculty of Medicine, Imperial College London, UK
[2]Imperial Healthcare NHS Trust, London, UK
[3]Department of Primary Education, School of Education, University of Ioannina, Greece
[4]Institute of Social and Preventive Medicine (ISPM), University of Bern, Finkenhubelweg 11, 3012 Bern, Switzerland
[5]Department of Obstetrics and Gynaecology, University of Helsinki and Helsinki University Hospital, Finland
[6]Department of Health Policy, London School of Economics & Political Science, UK
[7]Department of Gynaecologic Oncology, Lancashire Teaching Hospitals, Preston, UK
[8]Department of Obstetrics & Gynaecology, University of Ioannina and University Hospital of Ioannina, Greece

AA: a.athanasiou@imperial.ac.uk; AAV: a.veroniki@imperial.ac.uk; OE: oremiou@gmail.com; IK: i.kalliala@imperial.ac.uk; HN: h.naci@lse.ac.uk; SL: s.lever@imperial.ac.uk; MP: m.paraskevaidi@imperial.ac.uk; PMH: pierre.martin-hirsch@lthtr.nhs.uk; PB: p.bennett@imperial.ac.uk; EP: eparaske@cc.uoi.gr; GS: georgia.salanti@ispm.unibe.ch; MK: m.kyrgiou@imperial.ac.uk

Corresponding author:
Maria Kyrgiou, MSc, PhD, MRCOG
3rd Floor, IRDB, Department of Surgery & Cancer
Imperial College London, Hammersmith Campus
Du Cane Road, W12 0NN, London
Email: m.kyrgiou@imperial.ac.uk - Tel: +44 2075942177

Word Count: 3345

For peer review only

ABSTRACT

Introduction: Local treatments for cervical intra-epithelial neoplasia (CIN) and micro-invasive disease remove or ablate a cone-shaped part of the uterine cervix containing the abnormal cells. A trend towards less radical techniques has raised concerns that this may adversely impact the rates of precancerous and cancerous recurrence. However, there has been no strong evidence to support such claims. We hereby describe a protocol of a systematic review and network meta-analysis that will update the evidence and compare all relevant treatments in terms of efficacy and complications.

Methods and Analysis: Literature searches in electronic databases (CENTRAL, MEDLINE, EMBASE) or trial registries will identify published and unpublished randomised controlled trials (RCTs) and cohort studies comparing the efficacy and complications amongst different excisional and ablative techniques. The excisional techniques include cold knife, laser or fischer cone, large loop or needle excision of the transformation zone and the ablative radical point diathermy, cryotherapy, cold coagulation or laser ablation. The primary outcome will be residual/recurrent disease defined as abnormal histology or cytology of any grade, while secondary outcomes will include treatment failure rates defined as high-grade histology or cytology, histologically-confirmed CIN1+ or histologically-confirmed CIN2+, HPV positivity rates, involved margins rates, bleeding and cervical stenosis rates. We will assess the risk of bias in RCTs and observational studies using tools developed by the Cochrane Collaboration. Two authors will independently assess study eligibility, abstract the data, and assess the risk of bias. Random-effects meta-analyses and network meta-analyses will be conducted using the odds ratio for dichotomous outcomes and the mean difference for continuous outcomes. The quality of the evidence for the primary outcome will be assessed using the CINEMA tool.

Ethics and dissemination: Ethical approval not required. We will disseminate findings to clinicians, policy makers, patients and the public.

PROSPERO registration number: CRD42018115508

ARTICLE SUMMARY

Strengths and limitations of this study

- We plan to conduct the first network meta-analysis to assess the relative efficacy and complication rates of treatment methods for cervical pre-invasive and early micro-invasive disease.
- This study will produce comprehensive summaries of the clinical ranking of treatments and will employ methodologies that will allow the use of both randomised and observational data, aiming to utilise all published evidence.
- The results will inform clinicians, patients and clinical guidelines and will allow effective patient counselling at colposcopy clinics.
- We expect to find retrospective observational studies at high risk of recall, selection and publication bias. We will try to overcome this limitation by employing methods that aim to minimise bias.

For peer review only

KEYWORDS
"Cervical Intraepithelial Neoplasia/surgery"[Mesh]
"Cervical Intraepithelial Neoplasia/therapy"[Mesh]
"Treatment Outcome"[Mesh]
"Recurrence"[Mesh]
"Conization/adverse effects"[Mesh]
"Ablation Techniques/adverse effects"[Mesh]

INTRODUCTION

Organised screening programmes in countries such as the UK, have led to a dramatic decrease in the incidence and mortality from cervical cancer, especially when compared with the corresponding statistics for the other major cancers. Over a twenty-year period, from 1993-1995 to 2013-2015, the overall age-standardised incidence rate of cancer in females increased by 16% in the UK[1], whereas the corresponding data for cervical cancer showed a decrease of 24%[2]. Cervical cancer is largely preventable through detection and treatment of the pre-invasive precursor, cervical intra-epithelial neoplasia (CIN)[3]. The local treatment methods are divided into excisional and destructive (ablative) that aim to remove or ablate respectively a cone shaped part of the cervix that contains the 'transformation zone' with the precancerous cells. Although large loop excision of the transformation zone (LLETZ) is the most commonly used methods in the UK[4] given its ease of execution and low cost, the preference of techniques varies across Europe and internationally.

A Cochrane systematic review of RCTs concluded that all local treatment techniques are highly effective in preventing pre-invasive recurrence[5]. However, this review found no evidence of difference in treatment failure rates amongst the treatment techniques. This could be because the RCTs, and the subsequent meta-analysis might have been underpowered to detect differences between the treatments. The largest study recruited only 390 participants[6], while the majority of the rest were much smaller. A larger population-based study from Sweden[7], which included 150,883 women diagnosed and treated for CIN3 (3,148,222 woman-years), reported a doubled standardized incidence ratio for post-treatment invasive recurrence during the follow-up period of around 4 decades in comparison to the general population, and initiated debates on the impact that less radical treatments may have on the subsequent risk of invasion[8]. The trend towards techniques that remove smaller parts of the cervix can be attributed to the fact that many of these are easy to do, they are of low cost, and can be performed in an outpatient setting. Increased awareness of the impact of the more radical or deeper techniques on the risk of prematurity may have also contributed[9-20].

The impact of different techniques on the risk of pre-invasive and/or invasive recurrence remains therefore unclear. With some advocating the minimum radicality of treatment to prevent treatment-induced reproductive morbidity[10 21], and others raising concerns about the increase in the risk of future invasion[7 8], a definite answer regarding the relative merits and risks among the various treatment strategies is required.

For peer review only - http://bmjopen.bmj.com/site/about/guidelines.xhtml

Traditionally, treatment strategies are evaluated via large, expensive trials. Given the possibly comparable (and high) efficacy of most interventions for CIN, it is unlikely that any adequately powered RCT assessing the relative efficacy of different treatment techniques will ever be conducted. Such a trial would require thousands of women in order to reach the statistical power needed to detect differences in the pre-invasive and invasive recurrence rates. In summary, there is currently a lack of adequately powered randomised evidence to allow us to compare the various interventions. However, there is a plethora of available observational studies in the field. These studies are a potentially valuable source of evidence, and may act as a complement to the available randomised evidence, allowing us to more accurately assess the comparative effectiveness and safety of the various treatment alternatives. In this paper, we aim to perform a systematic review of both randomised and observational studies in the field, and quantitatively synthesize their findings in meta-analyses.

Systematic reviews and pairwise meta-analyses are widely accepted as a useful tool in comparative effectiveness research, and are commonly used to summarise, critically appraise and synthesise evidence from multiple studies. Investigators aiming to address a research question identify all relevant studies, evaluate their quality, synthesise their findings (meta-analysis) and interpret the provided evidence. Systematic reviews and meta-analyses have played a key role in providing evidence on the efficacy and safety of treatment methods and management strategies in cervical cancer prevention. However, the increased number of management strategies and multiple treatment options requires the use of more advanced evidence-synthesis methods.

Network meta-analysis (NMA) is an extension of pairwise meta-analysis, for the case when multiple treatments are available for the same condition. NMA has been recognised by the National Institute of Clinical Excellence (NICE)[22] and several international Health Technology Assessment (HTA) agencies[23 24] as a methodological tool that has the potential to increase precision in treatment effect estimates but also to infer on the clinical efficacy/ safety between treatments that have never been compared in trials. NMA uses both direct evidence (i.e. coming from studies comparing head-to-head the treatments of interest) and indirect evidence (i.e. coming from studies comparing the treatments of interest via an intermediate common comparator)[25-28], allows the estimation of relative treatment effects between all available interventions, and provides a clinically useful ranking of the different competing treatments. The methodology of NMA has never been used before to assess the comparative efficacy and complications of different treatment techniques used in the management of CIN. Furthermore, novel NMA methodologies will be employed to allow the use of both randomised and observational data.

The aim of this systematic review and NMA is to compare and clinically rank the alternative treatment techniques for CIN based on their efficacy, complications and adverse effects. This NMA forms part of the CIRCLE project (**C**ervical Cancer **I**ncidence, C**I**N **R**ecurrence and **R**eproduction after **L**ocal **E**xcision), which aims to generate a clinically useful raking of

For peer review only - http://bmjopen.bmj.com/site/about/guidelines.xhtml

alternative options for treatment of CIN according to their efficacy (risk of pre-invasive and invasive recurrence), morbidity and cost-effectiveness.

METHODS AND ANALYSIS

This protocol is written in accordance with the Preferred Reporting Items for Systematic Review and Meta-Analysis Protocols (PRISMA-P) (see Supplementary File 1)[29]. PROSPERO registration will be updated if we make any amendments to this protocol. The start date was 1st of October 2018 with expected end date 1st of October 2020.

Eligibility criteria of studies

Types of participants

We will include women of all ages treated with local surgical treatment for CIN (or glandular intraepithelial neoplasia) or micro-invasive early cervical cancer (stage IA1). We will only include women with histological diagnosis of CIN on punch biopsy or cone.

Types of interventions

The treatment techniques for cervical intraepithelial neoplasia (CIN) are divided into excisional and ablative. The excisional include cold knife conisation (CKC), laser conisation (LC), needle excision of the transformation zone (NETZ), also known as straight wire excision of the transformation zone (SWETZ), large loop excision of the transformation zone (LLETZ), also known as loop electrosurgical excisional procedure (LEEP) and Fischer cone biopsy excisor (FCBE), while the ablative include radical point diathermy (RD), cryotherapy (CT), cold coagulation (CC) and laser ablation (LA). Figure 1 displays a network example of comparisons between studied treatment techniques. When the treatment is not specified, we will group these under wider categories excision (ENS) or ablation (ANS).

Outcome measures

Primary outcome

- Treatment failure rates defined as any abnormal cytology [ASCUS (atypical squamous cells of undetermined significance) or worse] or histology (CIN1 or worse)

Secondary outcomes

- Treatment failure rates defined as high-grade abnormal cytology [HSIL (high-grade squamous intraepithelial lesion) or worse] or histology (CIN2 or worse)
- Treatment failure rates defined as residual or recurrent histologically-proven CIN1 or worse
- Treatment failure rates defined as residual or recurrent histologically-proven CIN2 or worse
- HPV positivity rates
- Involved margins rates (incomplete excision of the lesion): both, endocervical, ectocervical involvement
- Peri-operative or post-operative bleeding
- Cervical stenosis

For peer review only - http://bmjopen.bmj.com/site/about/guidelines.xhtml

Primary and secondary outcomes were chosen by clinical experts of the team. Treatment failure rates and HPV positivity rates will be reported at 6 to 12 months intervals based on the available data and reported intervals in the included studies.

Types of studies

We will include RCTs, quasi-RCTs and observational cohort studies comparing rates of treatment failure (recurrent/residual disease) or complications amongst the abovementioned surgical techniques. Single-arm studies not presenting a comparison will be excluded. Studies will be considered regardless of time or language.

Information sources and search strategy

The Cochrane Gynaecological Cancer Specialised Register, Cochrane Central Register of Controlled Trials (CENTRAL), MEDLINE and EMBASE will be searched for eligible studies by an experienced librarian, as presented in Supplementary File 2. Metaregister, Physicians Data Query, www.controlled-trials.com/rct, www.clinicaltrials.gov, www.cancer.gov/clinicaltrials and WHO Registry Network (https://www.who.int/ictrp/network/en/) will be searched for ongoing studies. ZETOC (http://zetoc.mimas.ac.uk) and WorldCat Dissertations will be searched for conference proceedings and theses, respectively. References of the retrieved articles and meta-analyses will be hand-searched, the "related articles" feature in MEDLINE will be employed and experts in the field will be contacted in an attempt to identify further reports of studies. Corresponding authors will be contacted for any relevant ongoing trials and unpublished data.

We will include both published and unpublished data and there will be no time, place or language restriction; articles in language other than English will be translated using online translation services.

Study selection

Two team members will independently screen titles and abstracts of citations at level 1, using the reference management software Zotero. At level 2, the full text of all potentially eligible articles will be assessed using the same inclusion criteria. Disagreements will be resolved through discussion with a third review author.

Data collection

Data from the included studies will be abstracted at level 3 by two reviewers independently using an a priori developed data collection form in Excel. The following data will be abstracted from the included studies: study characteristics, including author, publication year, country, study design, inclusion/exclusion criteria, and intervention details, participant characteristics, including age, CIN grade and smoking, and dropout rates, and outcome characteristics. In RCTs, we will prefer arm-level data (number of events and sample size per intervention arm for dichotomous data, and mean and standard deviation (SD) per intervention arm for continuous data), but if these are missing, the study-level data will be used in the analysis, e.g. reported odds ratios (ORs) for dichotomous outcomes and mean

For peer review only

differences (MDs) for continuous outcomes, along with a measure of uncertainly (e.g., confidence interval [CI]). For continuous outcome data not reported as means and standard deviations, we will first contact the corresponding study authors for further information, but if no additional data are provided, we will perform imputation methods to derive approximate effect measures[30 31]. When an eligible study is observational, we will prefer adjusted treatment effect estimates accounting for the impact of potential confounders, but if these are missing, the unadjusted estimated treatment effects will be abstracted with a corresponding uncertainty measure (e.g. CI). Disagreements will be resolved through consensus or the involvement of a third reviewer.

Risk of bias assessment

RCTs will be assessed for quality and risk of bias using the Cochrane risk of bias tool[32] in the following domains: randomisation process, deviations from the intended interventions, missing outcome data, measurement of the outcome and selection of the reported result. The risk of bias in each domain, as well as the overall risk of bias, will be rated as "low risk", "some concerns", or "high risk", after answering the signalling questions of each domain with "Yes", "Potentially Yes", "Potentially No" or "No". Non-randomised studies (NRS) will be assessed using the ROBINS-I tool[33] with potential confounding factors: grade of treated CIN, age and smoking. The following domains will be assessed for NRS: confounding, selection of participants into the study, classification of interventions, deviations from intended interventions, missing data, measurement of outcomes and selection of the reported results. The risk of bias in each domain, as well as the overall risk of bias, will be rated as "low", "moderate", "serious", or "critical", after answering the signalling questions of each domain with "Yes", "Potentially Yes", "Potentially No" or "No". Pairs of team members will independently assess the methodological quality and risk of bias of the eligible studies. Conflicts will be resolved through discussion or with a third investigator. When inadequate information is available from the studies to rate a risk of bias item, we will contact the corresponding study authors for clarification.

Statistical synthesis

Characteristics of included studies and Network

For each outcome, we will produce a network plot (see for example Figure 1) of the available evidence, as well as descriptive statistics, including comparison type, publication year, study design, outcome data, and potential effect modifiers (e.g., age).

Pairwise Meta-analyses

A random-effects meta-analysis will be conducted for each pairwise comparison in each outcome using the inverse variance model and the the Hartung-Knapp-Sidik-Jonkman method to estimate each summary treatment effect and its 95% CI[34-36]. The between-study variance will be estimated with the restricted maximum likelihood estimator, whereas its 95% CI with the Q-profile approach[34 37 38]. We will also use the I^2 statistic along a 95% CI[39 40] to evaluate between-study heterogeneity. For continuous outcomes we will report the summary MDs, whereas for dichotomous outcomes we will use the summary ORs, along with a 95% CI. The *metafor* package[41] in R[42] will be used for all meta-analyses.

For peer review only - http://bmjopen.bmj.com/site/about/guidelines.xhtml

Network meta-analyses (NMA)

Data synthesis

A random-effects NMA will be conducted, since we anticipate methodological and clinical between-study heterogeneity. We will assume common between-study variance parameter across treatment comparisons in the network, so that comparisons informed by a single study can borrow strength from the remaining network [43 44]. This assumption is clinically reasonable because all treatments included in the network of trials are of the same nature. The between-study variance will be estimated with the DerSimonian and Laird method of moments approach[45]. We will employ NMA models that account for different propensity of bias across different study designs as described in Efthimiou et al[46]. We will explore the impact of assigning different levels of credibility and subsequently down-weight the NRS according to experts' opinion and the results of the ROBINS-I tool in several sensitivity analyses.

Similar to the pairwise meta-analysis, for continuous outcomes we will report the estimated MDs, whereas for dichotomous outcomes we will use the estimated ORs, with a 95% CI. Along the 95% CI for the summary effect size, we will report 95% prediction intervals, i.e. the intervals within which the true underlying treatment effect is expected to lie in a new trial[47]. To rank the efficacy for each intervention, we will calculate the ranking probabilities for all treatments, the surface under the cumulative ranking curve (SUCRA) or P-scores, and the mean ranks[48 49]. A rank-heat plot will be used to depict the SUCRA values or P-scores across all outcomes[50]. We will apply all NMA models in R[42] using the *netmeta* package[51] and *rjags*[52] package.

Assessment of the transitivity assumption

One of the prerequisite assumptions in NMA is the transitivity assumption, under which the effect modifiers have a similar distribution across treatment comparisons in a network[27 53 54]. For the participants characteristics that are described in the inclusion criteria of our systematic review (section type of participants), it is reasonable to assume that all treatments we plan to compare (section type of interventions) are "jointly randomisable". That means that any patient that fulfils that inclusion criteria, could potentially be assigned to any of the interventions. Potential effect modifiers expected to influence the estimated treatment effects include year of study, level of income of study country (as defined by World Bank[55]), method of ascertainment of exposure/outcome (hospital records, registries or interviews/questionnaires), age, smoking and grade of CIN. For each pairwise comparison with available direct evidence we will summarize these characteristics and will visually inspect the similarity of the identified studies. We will also investigate the inclusion and exclusion criteria of all studies, to make sure that patients, treatments and outcomes in the studies are sufficiently similar in all aspects that might modify relative treatment effects. More specifically, we will compare the patient characteristics (such as severity, age, parity etc) across the different treatments. If these characteristics are found to have a similar distribution across treatments then transitivity is supported. If differences are found, then these will be addressed in subgroup and sensitivity analyses.

For peer review only - http://bmjopen.bmj.com/site/about/guidelines.xhtml

Assessment of statistical inconsistency

Consistency in a network of trials will be evaluated both locally and globally. We will first assess the consistency assumption locally by separating the direct from the indirect evidence for every comparison in a network to make judgements about their statistical differences, using the back-calculation method[56]. Then we will assess consistency in each network globally using the design-by-treatment interaction model[57]. We will conceptually explore for potential intransitivity in every network even in the absence of evidence for inconsistency, since the inconsistency tests have low power to detect true inconsistency[58 59]. If no substantial inconsistency is identified in the network of RCTs, we will then evaluate the agreement between RCTs and NRS using the same local and global approaches. Both local (back-calculation method) and global (design-by-treatment interaction model) assessments will be performed under the random-effects model in R[42] using the *netmeta* package[51].

In the NMA including both RCTs and NRS, we will assess for differences between the different study designs[46]. For each treatment comparison we will summarize evidence by up to four different types: direct randomised, indirect randomised, direct non-randomised and indirect non-randomised. If important discrepancies between these types are found, these will be investigated to confirm that the transitivity assumption holds (e.g. when randomised and non-randomised evidence are very different in terms of populations, interventions, etc., the transitivity assumption may be violated). If disagreement occurs for a certain characteristic, this will be explored through a network meta-regression model[26].

Exploring heterogeneity and inconsistency: subgroup analyses and meta-regression

The between-study heterogeneity will be explored by comparing the estimated between-study variance with the empirical distribution derived by Rhodes et al for continuous data[60] and the one derived by Turner et al for dichotomous data[61]. We will also compare 95% CIs with the 95% prediction intervals to infer on the magnitude of the between-study variance.

If at least 10 studies are available, the following potential sources of heterogeneity and/or inconsistency will be explored for the primary outcome using subgroup or meta-regression analyses: year of study, level of income of study country (as defined by World Bank[55]), method of ascertainment of exposure/outcome (hospital records, registries or interviews/questionnaires), age, smoking, grade of CIN, and disease severity (e.g., women treated for high-grade CIN, exclusion of cases of microinvasion). In order to minimise potential bias due to confounding from NRS (e.g. type of treatment or outcome affected by severity), we will also perform a sensitivity analysis excluding NRS without adjusted effect estimates.

Reporting bias and small study effects

We will assess small-study effects by visually exploring the funnel-plot for each treatment, and the comparison adjusted funnel plot[62] when at least 10 studies are available. We will also conduct a network meta-regression using the study variance as a covariate[63 64].

For peer review only - http://bmjopen.bmj.com/site/about/guidelines.xhtml

Assessment of the credibility of the evidence

For the primary outcome, two team members will determine the degree of confidence in the estimated NMA results using CINEMA[65] and the relevant online tool (http://cinema.ispm.ch/). The six CINEMA domains, within-study bias (i.e., risk of bias in the included studies), across-study bias (i.e., publication and reporting bias), indirectness, imprecision, heterogeneity and incoherence (i.e., differences between direct and indirect evidence)[65], will first be rated as high quality, and then they will be downgraded if judged appropriate to moderate, low, or very low quality.

Patient and Public involvement

Patients and the wider public have been involved from the design of this proposal through clinics and the Jo's Cervical Cancer Trust. They have assisted study design and to formulate the research questions. Their involvement will continue throughout the study on regular 6 monthly meetings and will guide the priority questions to be addressed, the development of research reports in lay language and the dissemination of the results.

ETHICS AND DISSEMINATION

We do not require ethical approval for this review. We aim to disseminate the results to clinicians, academic researchers, health agencies and decision makers, to patients and the public. We will publish the results in high impact open access journals and disseminate findings though presentations at medical conferences. The data will become available in public repositories. We will develop information sheets and briefings, highlighting the key findings and circulate newsletters. We will work closed with the Jo's Trust, charity in cervical cancer that frequently organises events to educate the public and also engage the media with interviews. We circulate findings in the Imperial College webpage and will circulate newsletters.

Figure 1. Network of possible pairwise comparisons between eligible treatment methods
Abbreviations: **FCBE**=Fischer cone biopsy excision; **LLETZ**= large loop excision of the transformation zone, also known as **LEEP**=loop electrosurgical excisional procedure; **NETZ**=needle excision of the transformation, also known as **SWETZ**=straight wire excision of the transformation zone; **LC**=laser conisation; **CKC**=cold knife conisation; **LA**=laser ablation; **CC**=cold coagulation; **CT**=cryotherapy; **RD**=radical point diathermy

REFERENCES

1. CancerResearchUK. Cancer incidence for all cancers combined 2018 [Available from: https://www.cancerresearchuk.org/health-professional/cancer-statistics/incidence/all-cancers-combined.
2. CancerResearchUK. Cervical cancer incidence statistics 2018 [Available from: https://www.cancerresearchuk.org/health-professional/cancer-statistics/statistics-by-cancer-type/cervical-cancer/incidence.
3. Scarinci IC, Garcia FA, Kobetz E, et al. Cervical cancer prevention: new tools and old barriers. *Cancer* 2010;116(11):2531-42. doi: 10.1002/cncr.25065 [published Online First: 2010/03/24]

For peer review only

4. NHS. Colposcopy: Treatments 2017 [Available from: https://www.nhs.uk/conditions/colposcopy/treatment/.
5. Martin-Hirsch PP, Paraskevaidis E, Bryant A, et al. Surgery for cervical intraepithelial neoplasia. *Cochrane Database Syst Rev* 2013(12):Cd001318. doi: 10.1002/14651858.CD001318.pub3 [published Online First: 2013/12/05]
6. Mitchell MF, Tortolero-Luna G, Cook E, et al. A randomized clinical trial of cryotherapy, laser vaporization, and loop electrosurgical excision for treatment of squamous intraepithelial lesions of the cervix. *Obstetrics and gynecology CNO - CN-00156163* 1998;92(5 XMAN - MEDLINE 1999008792 XMO - SU XSR - SR-GYNAECA):737-44.
7. Strander B, Hallgren J, Sparen P. Effect of ageing on cervical or vaginal cancer in Swedish women previously treated for cervical intraepithelial neoplasia grade 3: population based cohort study of long term incidence and mortality. *BMJ* 2014;348:f7361. doi: 10.1136/bmj.f7361 [published Online First: 2014/01/16]
8. Arbyn M, Kyrgiou M, Gondry J, et al. Long term outcomes for women treated for cervical precancer. *Bmj* 2014;348:f7700. doi: 10.1136/bmj.f7700 [published Online First: 2014/01/16]
9. Kyrgiou M, Koliopoulos G, Martin-Hirsch P, et al. Obstetric outcomes after conservative treatment for intraepithelial or early invasive cervical lesions: systematic review and meta-analysis. *Lancet* 2006;367(9509):489-98. doi: 10.1016/s0140-6736(06)68181-6 [published Online First: 2006/02/14]
10. Arbyn M, Kyrgiou M, Simoens C, et al. Perinatal mortality and other severe adverse pregnancy outcomes associated with treatment of cervical intraepithelial neoplasia: meta-analysis. *Bmj* 2008;337:a1284. doi: 10.1136/bmj.a1284 [published Online First: 2008/09/20]
11. Kyrgiou M, Mitra A, Arbyn M, et al. Fertility and early pregnancy outcomes after treatment for cervical intraepithelial neoplasia: systematic review and meta-analysis. *Bmj* 2014;349:g6192. doi: 10.1136/bmj.g6192 [published Online First: 2014/10/30]
12. Kyrgiou M, Mitra A, Arbyn M, et al. Fertility and early pregnancy outcomes after conservative treatment for cervical intraepithelial neoplasia. *Cochrane Database Syst Rev* 2015(9):Cd008478. doi: 10.1002/14651858.CD008478.pub2 [published Online First: 2015/09/30]
13. Kyrgiou M, Athanasiou A, Paraskevaidi M, et al. Adverse obstetric outcomes after local treatment for cervical preinvasive and early invasive disease according to cone depth: systematic review and meta-analysis. *Bmj* 2016;354:i3633. doi: 10.1136/bmj.i3633 [published Online First: 2016/07/30]
14. Kyrgiou M, Athanasiou A, Kalliala IEJ, et al. Obstetric outcomes after conservative treatment for cervical intraepithelial lesions and early invasive disease. *Cochrane Database Syst Rev* 2017;11:Cd012847. doi: 10.1002/14651858.Cd012847 [published Online First: 2017/11/03]
15. Kyrgiou M, Arbyn M, Martin-Hirsch P, et al. Increased risk of preterm birth after treatment for CIN. *Bmj* 2012;345:e5847. doi: 10.1136/bmj.e5847 [published Online First: 2012/09/07]
16. Castanon A, Landy R, Brocklehurst P, et al. Risk of preterm delivery with increasing depth of excision for cervical intraepithelial neoplasia in England: nested case-control study. *BMJ* 2014;349:g6223. [published Online First: 2014/11/05]
17. Noehr B, Jensen A, Frederiksen K, et al. Depth of cervical cone removed by loop electrosurgical excision procedure and subsequent risk of spontaneous preterm delivery. *Obstet Gynecol* 2009;114(6):1232-8. doi: 10.1097/AOG.0b013e3181bf1ef2 [published Online First: 2009/11/26]

For peer review only - http://bmjopen.bmj.com/site/about/guidelines.xhtml

18. Khalid S, Dimitriou E, Conroy R, et al. The thickness and volume of LLETZ specimens can predict the relative risk of pregnancy-related morbidity. *Bjog* 2012;119(6):685-91. doi: 10.1111/j.1471-0528.2011.03252.x [published Online First: 2012/02/15]
19. Founta C, Arbyn M, Valasoulis G, et al. Proportion of excision and cervical healing after large loop excision of the transformation zone for cervical intraepithelial neoplasia. *Bjog* 2010;117(12):1468-74. doi: 10.1111/j.1471-0528.2010.02709.x [published Online First: 2010/09/16]
20. Kyrgiou M, Valasoulis G, Stasinou SM, et al. Proportion of cervical excision for cervical intraepithelial neoplasia as a predictor of pregnancy outcomes. *Int J Gynaecol Obstet* 2015;128(2):141-7. doi: 10.1016/j.ijgo.2014.07.038 [published Online First: 2014/12/03]
21. Paraskevaidis E, Kyrgiou M, Martin-Hirsch P. Have we dismissed ablative treatment too soon in colposcopy practice? *Bjog* 2007;114(1):3-4. doi: 10.1111/j.1471-0528.2006.01178.x [published Online First: 2007/01/20]
22. NICE. Developing NICE guidelines: the manual. Process and methods (PMG20). Reviewing research evidence 2017 [Available from: https://www.nice.org.uk/process/pmg20/chapter/reviewing-research-evidence.
23. Caldwell DM, Dias S, Welton NJ. Extending Treatment Networks in Health Technology Assessment: How Far Should We Go? *Value Health* 2015;18(5):673-81. doi: 10.1016/j.jval.2015.03.1792 [published Online First: 2015/08/25]
24. CADTH. Network Meta-Analysis 2015 [Available from: https://www.cadth.ca/network-meta-analysis.
25. Caldwell DM, Ades AE, Higgins JP. Simultaneous comparison of multiple treatments: combining direct and indirect evidence. *BMJ* 2005;331(7521):897-900. doi: 10.1136/bmj.331.7521.897
26. Salanti G, Marinho V, Higgins JP. A case study of multiple-treatments meta-analysis demonstrates that covariates should be considered. *J Clin Epidemiol* 2009;62(8):857-64. doi: 10.1016/j.jclinepi.2008.10.001 [published Online First: 2009/01/20]
27. Cipriani A, Higgins JP, Geddes JR, et al. Conceptual and technical challenges in network meta-analysis. *Ann Intern Med* 2013;159(2):130-7. doi: 10.7326/0003-4819-159-2-201307160-00008
28. Efthimiou O, Debray TP, van Valkenhoef G, et al. GetReal in network meta-analysis: a review of the methodology. *Res Synth Methods* 2016;7(3):236-63. doi: 10.1002/jrsm.1195 [published Online First: 2016/01/13]
29. Moher D, Shamseer L, Clarke M, et al. Preferred reporting items for systematic review and meta-analysis protocols (PRISMA-P) 2015 statement. *Syst Rev* 2015;4:1. doi: 10.1186/2046-4053-4-1 [published Online First: 2015/01/03]
30. Furukawa TA, Barbui C, Cipriani A, et al. Imputing missing standard deviations in meta-analyses can provide accurate results. *J Clin Epidemiol* 2006;59(1):7-10. doi: 10.1016/j.jclinepi.2005.06.006 [published Online First: 2005/12/20]
31. Higgins JP, Altman DG, Gotzsche PC, et al. The Cochrane Collaboration's tool for assessing risk of bias in randomised trials. *BMJ* 2011;343:d5928. doi: 10.1136/bmj.d5928 [published Online First: 2011/10/20]
32. Higgins JPT, Sterne JAC, Savović J, et al. A revised tool for assessing risk of bias in randomized trials. In: Chandler J, McKenzie J, Boutron I, et al., eds.: Cochrane Methods. Cochrane Database of Systematic Reviews 2016.
33. Sterne JA, Hernan MA, Reeves BC, et al. ROBINS-I: a tool for assessing risk of bias in non-randomised studies of interventions. *BMJ* 2016;355:i4919. doi: 10.1136/bmj.i4919 [published Online First: 2016/10/14]

For peer review only

34. Veroniki AA, Jackson D, Viechtbauer W, et al. Methods to estimate the between-study variance and its uncertainty in meta-analysis. *Res Synth Methods* 2016;7(1):55-79. doi: 10.1002/jrsm.1164 [published Online First: 2015/09/04]
35. Hartung J, Knapp G. A refined method for the meta-analysis of controlled clinical trials with binary outcome. *Stat Med* 2001;20(24):3875-89. [published Online First: 2002/01/10]
36. Sidik K, Jonkman JN. A simple confidence interval for meta-analysis. *Stat Med* 2002;21(21):3153-9. doi: 10.1002/sim.1262 [published Online First: 2002/10/11]
37. Langan D, Higgins JPT, Jackson D, et al. A comparison of heterogeneity variance estimators in simulated random-effects meta-analyses. *Res Synth Methods* 2018 doi: 10.1002/jrsm.1316 [published Online First: 2018/08/02]
38. Viechtbauer W. Confidence intervals for the amount of heterogeneity in meta-analysis. *Stat Med* 2007;26(1):37-52. doi: 10.1002/sim.2514 [published Online First: 2006/02/08]
39. Higgins JP, Thompson SG, Deeks JJ, et al. Measuring inconsistency in meta-analyses. *BMJ* 2003;327(7414):557-60. doi: 10.1136/bmj.327.7414.557 [published Online First: 2003/09/06]
40. Veroniki AA, Jackson D, Viechtbauer W, et al. Recommendations for quantifying the uncertainty in the summary intervention effect and estimating the between-study heterogeneity variance in random-effects meta-analysis. In: Chandler J, McKenzie J, Boutron I, et al., eds.: Cochrane Methods. Cochrane Database of Systematic Reviews 2015.
41. Viechtbauer W. Conducting Meta-Analyses in R with the metafor Package. 2010;36(3):48.
42. RDevelopmentCoreTeam. R: A language and environment for statistical computing. Vienna, Austria: R Foundation for Statistical Computing, 2013.
43. Salanti G, Higgins JP, Ades AE, et al. Evaluation of networks of randomized trials. *Stat Methods Med Res* 2008;17(3):279-301. doi: 10.1177/0962280207080643 [published Online First: 2007/10/09]
44. Higgins JP, Whitehead A. Borrowing strength from external trials in a meta-analysis. *Stat Med* 1996;15(24):2733-49. doi: 10.1002/(SICI)1097-0258(19961230)15:24<2733::AID-SIM562>3.0.CO;2-0
45. Jackson D, White IR, Riley RD. Quantifying the impact of between-study heterogeneity in multivariate meta-analyses. *Stat Med* 2012;31(29):3805-20. doi: 10.1002/sim.5453 [published Online First: 2012/07/06]
46. Efthimiou O, Mavridis D, Debray TP, et al. Combining randomized and non-randomized evidence in network meta-analysis. *Stat Med* 2017;36(8):1210-26. doi: 10.1002/sim.7223 [published Online First: 2017/01/14]
47. Riley RD, Higgins JP, Deeks JJ. Interpretation of random effects meta-analyses. *BMJ* 2011;342:d549. doi: 10.1136/bmj.d549 [published Online First: 2011/02/12]
48. Salanti G, Ades AE, Ioannidis JP. Graphical methods and numerical summaries for presenting results from multiple-treatment meta-analysis: an overview and tutorial. *J Clin Epidemiol* 2011;64(2):163-71. doi: 10.1016/j.jclinepi.2010.03.016 [published Online First: 2010/08/05]
49. Rucker G, Schwarzer G. Ranking treatments in frequentist network meta-analysis works without resampling methods. *BMC medical research methodology* 2015;15:58. doi: 10.1186/s12874-015-0060-8 [published Online First: 2015/08/01]
50. Veroniki AA, Straus SE, Fyraridis A, et al. The rank-heat plot is a novel way to present the results from a network meta-analysis including multiple outcomes. *J Clin*

For peer review only

Epidemiol 2016;76:193-9. doi: 10.1016/j.jclinepi.2016.02.016 [published Online First: 2016/03/05]
51. Rücker G, Krahn U, König J, et al. netmeta: Network Meta-Analysis using Frequentist Methods. 2019 [Available from: https://github.com/guido-s/netmeta http://meta-analysis-with-r.org.
52. Plummer M. rjags: Bayesian Graphical Models using MCMC. R package version 4-8 2018 [Available from: https://CRAN.R-project.org/package=rjags.
53. Salanti G. Indirect and mixed-treatment comparison, network, or multiple-treatments meta-analysis: many names, many benefits, many concerns for the next generation evidence synthesis tool. *Res Synth Methods* 2012;3(2):80-97. doi: 10.1002/jrsm.1037 [published Online First: 2012/06/11]
54. Jansen JP, Naci H. Is network meta-analysis as valid as standard pairwise meta-analysis? It all depends on the distribution of effect modifiers. *BMC Med* 2013;11:159. doi: 10.1186/1741-7015-11-159 [published Online First: 2013/07/04]
55. WorldBank. World Bank list of economies 2019 [Available from: http://databank.worldbank.org/data/download/site-content/CLASS.xls accessed May 2019.
56. Konig J, Krahn U, Binder H. Visualizing the flow of evidence in network meta-analysis and characterizing mixed treatment comparisons. *Stat Med* 2013;32(30):5414-29. doi: 10.1002/sim.6001 [published Online First: 2013/10/15]
57. White IR, Barrett JK, Jackson D, et al. Consistency and inconsistency in network meta-analysis: model estimation using multivariate meta-regression. *Res Synth Methods* 2012;3(2):111-25. doi: 10.1002/jrsm.1045
58. Veroniki AA, Mavridis D, Higgins JP, et al. Characteristics of a loop of evidence that affect detection and estimation of inconsistency: a simulation study. *BMC medical research methodology* 2014;14:106. doi: 10.1186/1471-2288-14-106 [published Online First: 2014/09/23]
59. Song F, Clark A, Bachmann MO, et al. Simulation evaluation of statistical properties of methods for indirect and mixed treatment comparisons. *BMC medical research methodology* 2012;12:138. doi: 10.1186/1471-2288-12-138 [published Online First: 2012/09/14]
60. Rhodes KM, Turner RM, Higgins JP. Predictive distributions were developed for the extent of heterogeneity in meta-analyses of continuous outcome data. *J Clin Epidemiol* 2015;68(1):52-60. doi: 10.1016/j.jclinepi.2014.08.012 [published Online First: 2014/10/12]
61. Turner RM, Davey J, Clarke MJ, et al. Predicting the extent of heterogeneity in meta-analysis, using empirical data from the Cochrane Database of Systematic Reviews. *Int J Epidemiol* 2012;41(3):818-27. doi: 10.1093/ije/dys041 [published Online First: 2012/03/31]
62. Chaimani A, Higgins JP, Mavridis D, et al. Graphical tools for network meta-analysis in STATA. *PLoS One* 2013;8(10):e76654. doi: 10.1371/journal.pone.0076654 [published Online First: 2013/10/08]
63. Chaimani A, Salanti G. Using network meta-analysis to evaluate the existence of small-study effects in a network of interventions. *Res Synth Methods* 2012;3(2):161-76. doi: 10.1002/jrsm.57 [published Online First: 2012/06/01]
64. Mavridis D, Efthimiou O, Leucht S, et al. Publication bias and small-study effects magnified effectiveness of antipsychotics but their relative ranking remained invariant. *J Clin Epidemiol* 2016;69:161-9. doi: 10.1016/j.jclinepi.2015.05.027 [published Online First: 2015/07/27]

For peer review only

65. Salanti G, Del Giovane C, Chaimani A, et al. Evaluating the quality of evidence from a network meta-analysis. *PLoS One* 2014;9(7):e99682. doi: 10.1371/journal.pone.0099682 [published Online First: 2014/07/06]

STATEMENTS

Declaration

The corresponding author had full access to all the data in the study and the final responsibility for the decision to submit for publication.
The senior author MK (the manuscript's guarantor) affirms that the manuscript is an honest, accurate, and transparent account of the study being reported and that no important aspects of the study have been omitted.

Author statement

The study was conceived and designed by MK, GS and EP. The protocol was drafted by AA, MK, AAV, OE, IK, GS and was revised critically for important intellectual content by all authors (AA, AAV, OE, IK, HN, SL, MP, PMH, PB, EP, GS, MK).

Acknowledgements

We thank patients and patient representatives for their contribution to designing this study and developing the research questions.

Funding statement

This work is supported by National Institute for Health Research (NIHR) Research for Patient Benefits (Grant Reference Number PB-PG-0816-20004, P67307). It is also supported by the British Society of Colposcopy Cervical Pathology Jordan/Singer Award (P47773) and the Imperial College Healthcare Charity (P47907)(MK). AAV is funded by the European Union's Horizon 2020 (No 754936). None of the funders have any influence on the study design; in the collection, analysis, and interpretation of data; in the writing of the report; and in the decision to submit the article for publication.

Competing interests statement

We have read and understood BMJ policy on declaration of interests and declare that we have no conflict of interests. All authors have completed the ICMJE uniform disclosure form at www.icmje.org/coi_disclosure.pdf and declare: no support from any organisation for the submitted work; no financial relationships with any organisations that might have an interest in the submitted work in the previous three years; no other relationships or activities that could appear to have influenced the submitted work**.**
Dr Kyrgiou has received travel and conference expenses, honoraria and consultancy fees for commercial companies (Inovio, MSD etc); these activities are not related to the project.

Data sharing

No additional data available.

For peer review only - http://bmjopen.bmj.com/site/about/guidelines.xhtml

Copyright statement

"The Corresponding Author has the right to grant on behalf of all authors and does grant on behalf of all authors, a worldwide licence to the Publishers and its licensees in perpetuity, in all forms, formats and media (whether known now or created in the future), to i) publish, reproduce, distribute, display and store the Contribution, ii) translate the Contribution into other languages, create adaptations, reprints, include within collections and create summaries, extracts and/or, abstracts of the Contribution, iii) create any other derivative work(s) based on the Contribution, iv) to exploit all subsidiary rights in the Contribution, v) the inclusion of electronic links from the Contribution to third party material where-ever it may be located; and, vi) licence any third party to do any or all of the above."

For peer review only

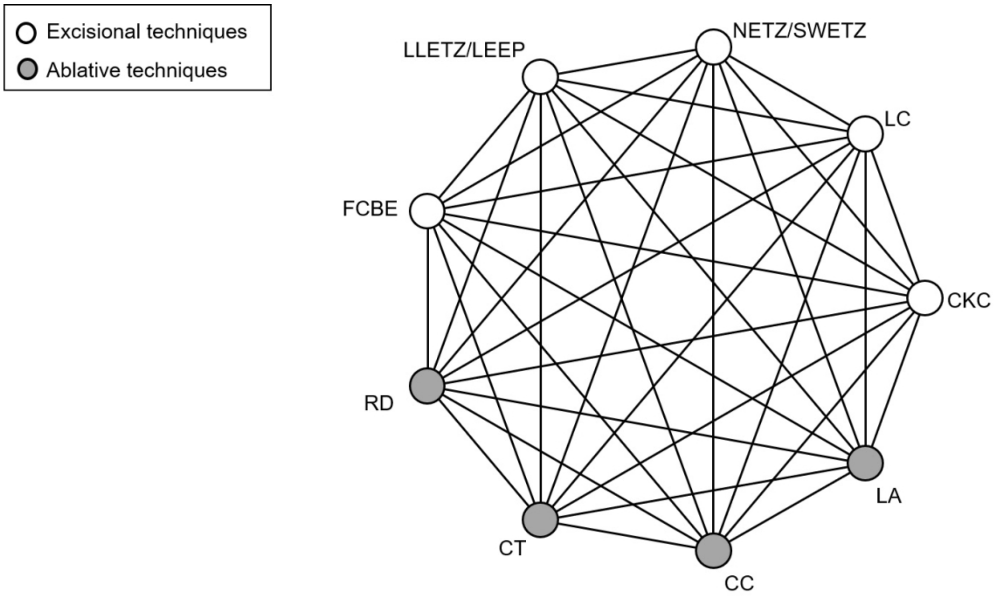

Network of possible pairwise comparisons between eligible treatment methods

117x71mm (300 x 300 DPI)

Supplementary File 1: Reporting checklist for protocol of a systematic review

Based on the PRISMA-P guidelines.

		Reporting Item	Page Number
Identification	#1a	Identify the report as a protocol of a systematic review	1
Update	#1b	If the protocol is for an update of a previous systematic review, identify as such	N/A (no previous NMA)
	#2	If registered, provide the name of the registry (such as PROSPERO) and registration number	2
Contact	#3a	Provide name, institutional affiliation, e-mail address of all protocol authors; provide physical mailing address of corresponding author	1
Contribution	#3b	Describe contributions of protocol authors and identify the guarantor of the review	14
	#4	If the protocol represents an amendment of a previously completed or published protocol, identify as such and list changes; otherwise, state plan for documenting important protocol amendments	4
Sources	#5a	Indicate sources of financial or other support for the review	15
Sponsor	#5b	Provide name for the review funder and / or sponsor	15
Role of sponsor or funder	#5c	Describe roles of funder(s), sponsor(s), and / or institution(s), if any, in developing the protocol	15
Rationale	#6	Describe the rationale for the review in the context of what is already known	3-4
Objectives	#7	Provide an explicit statement of the question(s) the review will address with reference to participants, interventions, comparators, and outcomes (PICO)	4
Eligibility criteria	#8	Specify the study characteristics (such as PICO, study design, setting, time frame) and report characteristics (such as years considered, language, publication status) to be used as criteria for eligibility for the review	5

For peer review only

Information sources	#9	Describe all intended information sources (such as electronic databases, contact with study authors, trial registers or other grey literature sources) with planned dates of coverage	5-6
Search strategy	#10	Present draft of search strategy to be used for at least one electronic database, including planned limits, such that it could be repeated	5; Supplementary File 2
Study records - data management	#11a	Describe the mechanism(s) that will be used to manage records and data throughout the review	6
Study records - selection process	#11b	State the process that will be used for selecting studies (such as two independent reviewers) through each phase of the review (that is, screening, eligibility and inclusion in meta-analysis)	6
Study records - data collection process	#11c	Describe planned method of extracting data from reports (such as piloting forms, done independently, in duplicate), any processes for obtaining and confirming data from investigators	6
Data items	#12	List and define all variables for which data will be sought (such as PICO items, funding sources), any pre-planned data assumptions and simplifications	6
Outcomes and prioritization	#13	List and define all outcomes for which data will be sought, including prioritization of main and additional outcomes, with rationale	5
Risk of bias in individual studies	#14	Describe anticipated methods for assessing risk of bias of individual studies, including whether this will be done at the outcome or study level, or both; state how this information will be used in data synthesis	6-7
Data synthesis	#15a	Describe criteria under which study data will be quantitatively synthesised	7
	#15b	If data are appropriate for quantitative synthesis, describe planned summary measures, methods of handling data and methods of combining data from studies, including any planned exploration of consistency (such as I2, Kendall's τ)	7-8
	#15c	Describe any proposed additional analyses (such as sensitivity or subgroup analyses, meta-regression)	8-9
	#15d	If quantitative synthesis is not appropriate, describe the type of	N/A

For peer review only

		summary planned	
Meta-bias(es)	#16	Specify any planned assessment of meta-bias(es) (such as publication bias across studies, selective reporting within studies)	9
Confidence in cumulative evidence	#17	Describe how the strength of the body of evidence will be assessed (such as GRADE)	9

The PRISMA-P checklist is distributed under the terms of the Creative Commons Attribution License CC-BY 4.0. This checklist can be completed online using https://www.goodreports.org/, a tool made by the EQUATOR Network in collaboration with Penelope.ai

For peer review only

Supplementary File 2: Search algorithms

Medline Ovid RCT only

1 exp Cervical Intraepithelial Neoplasia/
2 CIN.mp.
3 (cervi* and (intraepithel* or epithel*)).mp.
4 (cervi* and dysplasia).mp.
5 (cervi* and carcinoma in situ).mp.
6 (cervi* and cancer in situ).mp.
7 (cervi* and (precancer* or pre-cancer*)).mp.
8 1 or 2 or 3 or 4 or 5 or 6 or 7
9 surgery.fs.
10 exp Gynecologic Surgical Procedures/
11 (surg* or ablat* or excis* or cryotherapy or laser* or cone or conisation or biopsy or transformation zone or LLETZ or LEEP).mp.
12 9 or 10 or 11
13 8 and 12
14 randomized controlled trial.pt.
15 controlled clinical trial.pt.
16 randomized.ab.
17 placebo.ab.
18 clinical trials as topic.sh.
19 randomly.ab.
20 trial.ti.
21 14 or 15 or 16 or 17 or 18 or 19 or 20
22 13 and 21

Medline Ovid NON RCT only

1. exp Cervical Intraepithelial Neoplasia/
2. CIN.mp. [mp=title, abstract, original title, name of substance word, subject heading word, keyword heading word, protocol supplementary concept word, rare disease supplementary concept word, unique identifier, synonyms]
3. (cervi* and (intraepithel* or epithel*)).mp. [mp=title, abstract, original title, name of substance word, subject heading word, keyword heading word, protocol supplementary concept word, rare disease supplementary concept word, unique identifier, synonyms]
4. (cervi* and dysplasia).mp. [mp=title, abstract, original title, name of substance word, subject heading word, keyword heading word, protocol supplementary concept word, rare disease supplementary concept word, unique identifier, synonyms]

For peer review only

5. (cervi* and carcinoma in situ).mp. [mp=title, abstract, original title, name of substance word, subject heading word, keyword heading word, protocol supplementary concept word, rare disease supplementary concept word, unique identifier, synonyms]
6. (cervi* and cancer in situ).mp. [mp=title, abstract, original title, name of substance word, subject heading word, keyword heading word, protocol supplementary concept word, rare disease supplementary concept word, unique identifier, synonyms]
7. (cervi* and (precancer* or pre-cancer*)).mp. [mp=title, abstract, original title, name of substance word, subject heading word, keyword heading word, protocol supplementary concept word, rare disease supplementary concept word, unique identifier, synonyms]
8. 1 or 2 or 3 or 4 or 5 or 6 or 7
9. surgery.fs.
10. exp Gynecologic Surgical Procedures/
11. (surg* or ablat* or excis* or cryotherapy or laser* or cone or conisation or biopsy or transformation zone or LLETZ or LEEP).mp. [mp=title, abstract, original title, name of substance word, subject heading word, keyword heading word, protocol supplementary concept word, rare disease supplementary concept word, unique identifier, synonyms]
12. 9 or 10 or 11
13. 8 and 12
14. randomized controlled trial.pt.
15. controlled clinical trial.pt.
16. randomized.ab.
17. placebo.ab.
18. clinical trials as topic.sh.
19. randomly.ab.
20. trial.ti.
21. groups.ab.
22. exp cohort studies/
23. exp case-control studies/
24. (cohort* or prospective* or retrospective* or (case* and (control* or series))).mp.
25. 14 or 15 or 16 or 17 or 18 or 19 or 20 or 21 or 22 or 23 or 24
26. (animals not (humans and animals)).sh.
27. 25 not 26
28. 13 and 27

Embase Ovid RCT only

1 exp Uterine Cervix Carcinoma in Situ/

For peer review only

2 CIN.mp.
3 (cervi* and (intraepithel* or epithel*)).mp.
4 (cervi* and dysplasia).mp.
5 (cervi* and carcinoma in situ).mp.
6 (cervi* and cancer in situ).mp.
7 (cervi* and (precancer* or pre-cancer*)).mp.
8 1 or 2 or 3 or 4 or 5 or 6 or 7
9 su.fs.
10 exp gynecologic surgery/
11 (surg* or ablat* or excis* or cryotherapy or laser* or cone or conisation or biopsy or transformation zone or LLETZ or LEEP).mp.
12 9 or 10 or 11
13 8 and 12
14 crossover procedure/
15 double-blind procedure/
16 randomized controlled trial/
17 single-blind procedure/
18 random*.mp.
19 factorial*.mp.
20 (crossover* or cross over* or cross-over*).mp.
21 placebo*.mp.
22 (double* adj blind*).mp.
23 (singl* adj blind*).mp.
24 assign*.mp.
25 allocat*.mp.
26 volunteer*.mp.
27 14 or 15 or 16 or 17 or 18 or 19 or 20 or 21 or 22 or 23 or 24 or 25 or 26
28 13 and 27

Embase Ovid All Studies

1. exp Uterine Cervix Carcinoma in Situ/
2. CIN.mp. [mp=title, abstract, heading word, drug trade name, original title, device manufacturer, drug manufacturer, device trade name, keyword, floating subheading word]
3. (cervi* and (intraepithel* or epithel*)).mp. [mp=title, abstract, heading word, drug trade name, original title, device manufacturer, drug manufacturer, device trade name, keyword, floating subheading word]

For peer review only

4. (cervi* and dysplasia).mp. [mp=title, abstract, heading word, drug trade name, original title, device manufacturer, drug manufacturer, device trade name, keyword, floating subheading word]
5. (cervi* and carcinoma in situ).mp. [mp=title, abstract, heading word, drug trade name, original title, device manufacturer, drug manufacturer, device trade name, keyword, floating subheading word]
6. (cervi* and cancer in situ).mp. [mp=title, abstract, heading word, drug trade name, original title, device manufacturer, drug manufacturer, device trade name, keyword, floating subheading word]
7. (cervi* and (precancer* or pre-cancer*)).mp. [mp=title, abstract, heading word, drug trade name, original title, device manufacturer, drug manufacturer, device trade name, keyword, floating subheading word]
8. 1 or 2 or 3 or 4 or 5 or 6 or 7
9. su.fs.
10. exp gynecologic surgery/
11. (surg* or ablat* or excis* or cryotherapy or laser* or cone or conisation or biopsy or transformation zone or LLETZ or LEEP).mp.
12. 9 or 10 or 11
13. 8 and 12
14. exp controlled clinical trial/
15. randomized.ab.
16. randomly.ab.
17. trial.ab.
18. groups.ab.
19. exp cohort analysis/
20. cohort*.mp.
21. exp retrospective study/
22. exp prospective study/
23. (case* and series).mp.
24. 14 or 15 or 16 or 17 or 18 or 19 or 20 or 21 or 22 or 23
25. 13 and 24

CENTRAL
#1 MeSH descriptor Cervical Intraepithelial Neoplasia explode all trees
#2 CIN
#3 cervi* and (intraepithel* or epithel*)
#4 cervi* and dysplasia
#5 cervi* and carcinoma in situ
#6 cervi* and cancer in situ
#7 cervi* and (precancer* or pre-cancer*)
#8 (#1 OR #2 OR #3 OR #4 OR #5 OR #6 OR #7)

For peer review only

#9 Any MeSH descriptor with qualifier(s): [Surgery - SU]
#10 MeSH descriptor: [Gynecologic Surgical Procedures] explode all trees
#11 surg* or ablat* or excis* or cryotherapy or laser* or cone or conisation or biopsy or transformation zone or LLETZ or LEEP
#12 #9 or #10 or #11
#13 #8 and #12

For peer review only
